# Clinical glycoproteomics: methods and diseases

**DOI:** 10.1002/mco2.760

**Published:** 2024-10-04

**Authors:** Yujia Wang, Kaixin Lei, Lijun Zhao, Yong Zhang

**Affiliations:** ^1^ Department of General Practice Ward/International Medical Center Ward General Practice Medical Center and Institutes for Systems Genetics West China Hospital Sichuan University Chengdu China

**Keywords:** cancers, clinical glycoproteomics, glycosylation, kidney diseases, mass spectrometry, metabolic diseases

## Abstract

Glycoproteins, representing a significant proportion of posttranslational products, play pivotal roles in various biological processes, such as signal transduction and immune response. Abnormal glycosylation may lead to structural and functional changes of glycoprotein, which is closely related to the occurrence and development of various diseases. Consequently, exploring protein glycosylation can shed light on the mechanisms behind disease manifestation and pave the way for innovative diagnostic and therapeutic strategies. Nonetheless, the study of clinical glycoproteomics is fraught with challenges due to the low abundance and intricate structures of glycosylation. Recent advancements in mass spectrometry‐based clinical glycoproteomics have improved our ability to identify abnormal glycoproteins in clinical samples. In this review, we aim to provide a comprehensive overview of the foundational principles and recent advancements in clinical glycoproteomic methodologies and applications. Furthermore, we discussed the typical characteristics, underlying functions, and mechanisms of glycoproteins in various diseases, such as brain diseases, cardiovascular diseases, cancers, kidney diseases, and metabolic diseases. Additionally, we highlighted potential avenues for future development in clinical glycoproteomics. These insights provided in this review will enhance the comprehension of clinical glycoproteomic methods and diseases and promote the elucidation of pathogenesis and the discovery of novel diagnostic biomarkers and therapeutic targets.

## Graphical abstract

An overview of clinical glycoproteomics: methods and diseases. The primary types of glycosylations include N‐glycosylation and O‐glycosylation. Three main glycoproteomic strategies are employed, with the bottom‐up approach being the most commonly utilized. Alterations in the glycoproteome are intimately linked with the onset and progression of various diseases, such as brain diseases, cardiovascular diseases, cancers, kidney diseases, and metabolic diseases.

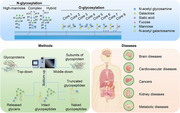

## 1 INTRODUCTION

**FIGURE 1:**
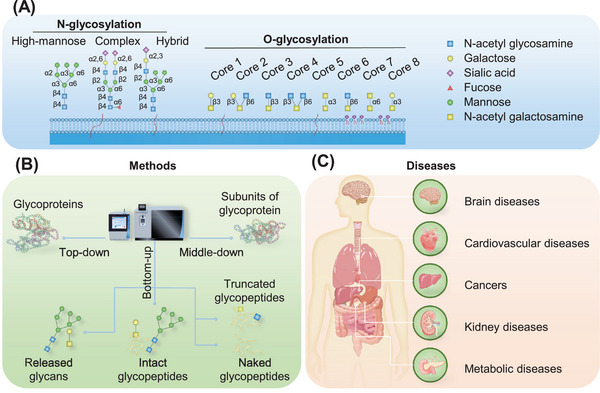
An overview of clinical glycoproteomics: methods and diseases. (A) The primary types of glycosylations include N‐glycosylation (encompassing high‐mannose, complex, and hybrid types) and O‐glycosylation (featuring eight core structures). (B) Three main glycoproteomic strategies (top‐down, middle‐down, and bottom‐up) are employed, with the bottom‐up approach being the most commonly utilized. (C) Alterations in the glycoproteome are intimately linked with the onset and progression of various diseases, such as brain diseases, cardiovascular diseases, cancers, kidney diseases, and metabolic diseases.

The Human Genome Project has made great contributions to unraveling the mysteries of life.^1^, ^2^ With the successful completion of the program, the life sciences entered a postgenome era, and the Human Proteome Project was launched.^3^, ^4^ Proteomics, as a core task of life science research in this era, involves studying the composition and changes in the proteomes of cells, tissues, or organisms at a holistic level, and clarifying the expression patterns and functional patterns of all proteins in organisms.^5^ However, posttranslational modifications (PTMs) increase the complexity of protein structure, enhance functionality, refine regulation, and increase specificity.^6^ Glycosylation, the most abundant and diverse form of PTM of proteins in eukaryotes, refers to the intricate process of attaching glycans to biomolecules (protein, lipid, and ribonucleic acid), a critical modification that can occur during or after the production of these essential biomolecules. It is catalyzed by glycosyltransferases and occurs predominantly in the endoplasmic reticulum (ER) and Golgi apparatus.^7^ More than half of the proteins in the human proteome are glycosylated, including United States (US) Food and Drug Administration (FDA)‐approved tumor biomarkers (such as prostate‐specific antigen [PSA] and alpha fetal protein [AFP]).^8^ Therefore, glycoproteins play crucial roles in various physiological and pathological processes, highlighting their importance in the complex network of biological systems.^9^ Protein glycosylation can be categorized into O‐glycosylation, N‐glycosylation, O‐GlcNAcylation, C‐mannosylation, glycosylphosphatidylinositol (GPI) anchoring, and so on. In this review, we focus on the two most commonly studied glycosylation. N‐glycosylation occurs on asparagine in the motif Asn–Xxx–Ser/Thr/Cys (with Xxx any amino acids different from proline) with three major N‐glycan types (high‐mannose‐type, hybrid‐type, complex‐type), and O‐glycosylation occurs on hydroxyl groups, mostly threonine and serine, with eight O‐glycan core structures (from core 1 to core 8) (Figure 1A).^10^ Diving deeper into the realm of glycosylation, glycoproteomics has emerged as a specialized subfield of proteomics that focuses on identifying and characterizing glycosylation events at the proteome scale.^11^ As a result, clinical glycoproteomics has emerged as a subfield of glycoproteomics.^12^ Herein, we define glycoproteomics as the integration of advanced glycoproteomic technology with other cutting‐edge technologies to analyze clinical samples, with the goal of finding abnormal expression of glycoproteins, glycopeptides, glycosites, and glycans. The objective of clinical glycoproteomics is to translate fundamental discoveries into clinical applications, leading to significant advancements in the prevention and treatment of various diseases.

Clinical glycoproteomic research faces numerous methodological challenges. The low abundance, complexity, micro‐ and macroheterogeneity, and high dynamic variability of protein glycosylation necessitate the use of liquid chromatography tandem mass spectrometry (LC–MS/MS) for site‐specific glycosylation characterization.^13^ Owing to its unparalleled advantages in the high‐throughput and high‐sensitivity identification of glycosylation, LC–MS/MS has emerged as an indispensable tool in the glycoproteomics toolkit.^14^ In recent decades, LC–MS/MS has not only demonstrated its value in the intricate analysis of glycoproteins but also cemented its role as an important tool in clinical diagnostics. Nonetheless, the field of clinical glycoproteomics demands enhancements in detection accuracy, sensitivity, the ability to perform in situ real‐time analysis, requirements for a smaller sample size and faster detection speed.^15^ Consequently, progress in clinical sample processing methods, advancements in mass spectrometry technologies, and improvements in analysis software are imperative. In recent years, significant efforts have been dedicated to developing related methods and technologies to increase the quality of glycoproteomic studies.^10^ There are three primary strategies for exploring the glycoproteome, each offering unique insights into the structure and function of glycoproteins (Figure 1B). First, the top‐down strategy is predominantly used for studying the whole glycoproteins, including recombinant glycoproteins and monoclonal antibodies (mAbs). It can present the general characteristics of the entire glycoproteins but cannot provide detailed information about glycosylation.^16^ Second, the middle‐down strategy narrows the focus to subunits of the glycoproteins or longer glycopeptides. Compared with top‐down strategy, it can confirm more complete sequences and glycoform locations.^17^ Third, the bottom‐up strategy is the most commonly used strategy in clinical glycoproteomics.^10^ By analyzing the glycans released by chemical or enzymatic methods, the composition and structural information of all the glycans on the glycoproteome can be obtained, but glycosite information can be lost. Although the analysis of naked glycopeptides or truncated glycopeptides can identify glycosites and the extent of glycan occupancy, it does not provide information about the native glycan composition and structure. Site‐specific glycosylation analysis is crucial for understanding the functional implications of glycosylation in glycoproteins. It can be implemented through the analysis of intact glycopeptides (IGPs; glycopeptides decorated with N/O‐glycans), so this method has become mainstream in clinical glycoproteomics. This review provides a comprehensive overview and discussion of advancements in the field of clinical glycoproteomics, particularly focusing on cutting‐edge methodologies for the analysis of site‐specific glycosylation or IGPs.^13^ Significant progress has been made in various aspects of the analytical process, including the preparation of clinical samples, the enrichment of IGPs, and the detection of IGPs through LC–MS/MS and specialized software. These advancements have revolutionized the glycoproteomics field, enabling researchers to identify hundreds to thousands of glycosites or IGPs in a single experiment.

Different types of glycosylation may play different roles in physiological and pathological processes.^18^ N‐glycosylation can affect protein folding, stability, solubility, immunogenicity, half‐life, and drug properties and is widely involved in cell recognition, ‌cell adhesion, cell transformation, regulation, ‌signaling, immune response, and other physiological processes.^19^ ‌O‐glycosylation plays indispensable roles in protein secretion, aggregation, phase separation, immunogenicity, and half‐life and is widely involved in several physiological functions, such as cell–cell interactions, transmembrane receptor activation, pathogen binding and immune cell modulation.^20^ Therefore, abnormal changes in protein N/O‐glycosylation are closely related to the occurrence and development of various diseases.^21^ The development of clinical glycoproteomic methods and technologies has promoted their application in various diseases, such as brain diseases, cardiovascular diseases (CVDs), cancers, kidney diseases, and metabolic diseases (Figure 1C). Through the meticulous examination of biochemical compounds present in human urine, blood, cerebrospinal fluid (CSF), pathological tissue, and other clinical samples, LC–MS/MS has facilitated breakthroughs in the diagnosis and understanding of various diseases. Its ability to deliver precise and comprehensive insights into the molecular underpinnings of health and disease highlights the indispensable role of LC–MS/MS in advancing both scientific research and clinical practice.^22^ Recent advancements have led to the application of glycoproteomic technology across the spectrum of clinical diagnostics and therapeutic interventions. This cutting‐edge approach has been pivotal in identifying disease biomarkers, exploring pathological metabolites, and developing vaccines that specifically target glycosylation processes.^23^, ^24^ For instance, in the context of kidney disease, research has revealed a significant connection between irregular glycosylation patterns and the progression of renal disorders. Specifically, aberrant glycosylation is intricately linked with the mechanisms that drive kidney diseases, highlighting the essential role of precise glycosylation patterns in preserving renal health and paving the way for targeted therapeutic strategies.^25^, ^26^, ^27^ In this review, we summarize and discuss the emerging trends within clinical glycoproteomics to illustrate how the field is moving away from studies that have focused on identifying glycosylated substrates to study specific mechanisms and disease states.

This review outlines the fundamental principles and logic underpinning of glycoproteomic technology and further investigates the current contributions of this cutting‐edge technology to the scientific field, emphasizing its capacity to transform different disease diagnoses and treatments. Through a comprehensive discussion, this review highlights the critical role of glycoproteomics in enhancing our comprehension and management of diverse diseases, ultimately leading to better patient outcomes. This exploration not only sheds light on the potential of glycoproteomics but also underscores its importance in the ongoing pursuit of medical advancement.

## 2 OVERVIEW OF CLINICAL GLYCOPROTEOMIC METHODS

### 2.1 Fundamental principles and logic underpinning of clinical glycoproteomic methods

**FIGURE 2:**
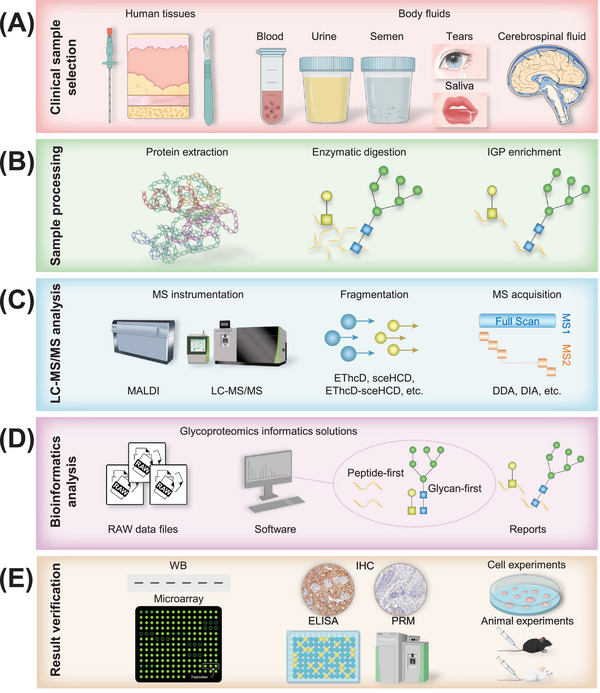
The methodology of clinical glycoproteomics. (A) Selection of clinical samples. This involves choosing the appropriate human tissues and body fluids for analysis. (B) Common sample processing workflow. The key steps include the extraction of proteins, enzymatic digestion, and IGP enrichment. (C) LC–MS/MS analysis. Critical considerations include MS instrumentation, fragmentation mode, and acquisition method. (D) Bioinformatics analysis. Solutions for data analysis and interpretation in the field of clinical glycoproteomics. (E) Verification of results. This stage encompasses molecular, cellular, and animal experiments to confirm the findings. IGP, intact glycopeptide; LC–MS/MS, liquid chromatography–tandem mass spectrometry; MALDI, matrix‐assisted laser desorption ionization; EThcD, electron‐transfer/higher‐energy collisional dissociation; sceHCD, stepped collision energy/higher‐energy collisional dissociation; DDA, data‐dependent acquisition; DIA, data‐independent acquisition; WB, western blot; ELISA, enzyme‐linked immunosorbent assay; IHC, immunohistochemistry; PRM, parallel reaction monitoring.

After glycosylation, functional glycoproteins are produced, which play crucial roles in biological systems. However, this modification process can sometimes result in the abnormal production of specific glycoproteins with abnormally altered glycosites and/or glycans in a pathological manner, which can then lead to a series of functional changes.^28^ The methodology of glycoproteomics involves several key steps: clinical sample selection, sample processing (protein extraction, enzymatic digestion, IGP enrichment), LC–MS/MS analysis, bioinformatics analysis, and result verification (Figure 2). The first crucial step in glycoproteomic analysis is ensuring the quality of clinical samples, as it directly impacts the final results. Clinical samples with greater complexity, heterogeneity, and lower glycoprotein concentrations present significant analytical challenges.^29^ Following sample processing, it is imperative to isolate and/or enrich glycoproteins and/or IGPs, a critical step owing to the limited abundance of glycosylation. The enrichment preference of IGPs may exist in different enrichment materials or strategies. This IGP enrichment process is often combined with LC–MS/MS technology, which has become essential in glycoproteomic research for in‐depth identification of IGP.^30^, ^31^ The selection of the mass spectrometer and fragmentation mode is crucial because of the varying fragmentation energy required for peptide and glycan chain fragmentation.^32^ This choice directly impacts the accuracy and/or depth of IGP identification. Annotating IGP MS/MS spectra is a challenging task that involves correctly assigning the peptide carrier, glycosite, and attached glycans. Numerous software programs and algorithms (Byonic, MSFraggerGlyco, pGlyco series, StrucGP, etc.) have been developed for the identification and quantitative analysis of glycoproteomics. Despite the existing bioinformatics hurdles in glycoproteomics, some tools have shown exceptional performance and great potential in offering more sensitive, accurate, and comprehensive glycosylation information.^33^, ^34^, ^35^ Moreover, LC–MS/MS‐based glycoproteomic findings should be validated by additional methods, such as small‐scale biochemical experiments and large‐scale cohort validation.^36^ Crucially, the functional role of glycoproteins should be confirmed through cell and animal experiments. In summary, the intricate nature of glycoproteomics underscores the complexity and accuracy required to decipher the enigmas of glycoproteins and their biological importance.

### 2.2 Clinical sample selection

Prior to conducting clinical glycoproteomics, it is imperative to meticulously deliberate on every facet of the study, encompassing experimental design, clinical specimens, equipment, and software.^15^ Notably, the selection of clinical samples (human tissues and body fluids) significantly impacts the credibility of the experimental findings (Figure 2A). In particular, the following aspects should be taken into account: disease subtypes and stages, clinical data, specimen type, sample categorization, sample volume, collection procedures, preprocessing methods, storage conditions, transportation logistics, and so on. For example, Cao et al.^37^ performed glycoproteomic analysis on pancreatic tissue samples from pancreatic ductal adenocarcinoma (PDAC) and normal adjacent tissue. They gathered clinical data and considered various tissue source sites and countries. Standardization was applied to the collection, pretreatment, storage, and transportation of the tissue samples. In clinical glycoproteomics, physiological and pathological tissue samples acquired through puncture and surgery, as well as body fluids (such as blood, urine, semen, tears, saliva, and CSF) obtained through clinical examinations, are the most commonly utilized sources (Figure 2A). Therefore, the experimental design should include an appropriate control group and experimental group, ensure a sufficient number of cases in each group (typically exceeding 30), a sufficient sample size (usually ensuring the extraction of at least 100 µg of protein), and minimize unnecessary contamination. Additionally, establishing standardized protocols for sample collection, pretreatment, storage, and transportation is crucial for conducting subsequent experiments effectively. It is essential to adhere strictly to these procedures to ensure the reliability and reproducibility of the results.

### 2.3 Sample processing

#### 2.3.1 Protein extraction

Clinical sample processing is an important bottleneck in glycoproteomic analysis. To reduce interference during experiments, it is essential to establish a standardized process tailored to the experimental goals and sample types. The initial step in clinical sample processing is protein extraction (Figure 2B). Depending on the experimental objectives, one must decide whether to extract all proteins or target specific glycoproteins (such as highly abundant immunoglobulin [Ig] and uromodulin). To effectively weigh the advantages and disadvantages of various extraction strategies for glycoproteomic analysis, we propose a comprehensive approach. Initially, people should meticulously optimize and compare several glycoproteomic extraction techniques, focusing on both the extraction reagents and the methodologies employed. Subsequently, the method that demonstrates superior efficiency and reliability is selected for the extraction of glycoproteomes from large‐scale clinical samples. In the final stage, based on the identification of glycoproteins of particular interest, people can tailor the extraction process to these target glycoproteins. This strategy ensures a balanced consideration of efficiency and specificity in the extraction process, facilitating a more precise and insightful analysis of the glycoproteome. Additionally, the choice of extraction solvent and method should align with the sample type. For instance, when processing the most commonly used clinical sample of human plasma, removing high‐abundance proteins (HAPs) is important. HAPs make up 85% of the total proteome in human plasma, with the concentration of plasma proteins spanning a dynamic range of over 10 orders of magnitude.^38^ These HAPs can cause MS signal suppression, limiting the detection of low‐abundance proteins (LAPs). Many methods have been developed to simplify and standardize plasma samples. Immuno‐based depletion techniques, such as targeting the top two or top 14 most abundant proteins, as well as the use of combinatorial peptide ligand libraries, are commonly employed in proteomics research.^39^ In recent decades, nanotechnology‐based sample processing strategies have become efficient methods for conducting automated plasma processing and in‐depth proteome profiling. Magnetic nanoparticles (MNs) are small in size and have large specific surface areas and abundant affinity sites, allowing for specific or nonspecific enrichment of proteins or PTMs.^40^ Upon entering the plasma, the MNs initially adsorb HAPs to form a protein corona at the nano‐plasma interface. These HAPs are subsequently replaced by LAPs with higher affinity.^41^ Urine samples are known for their ease of clinical collection, noninvasive nature, and ability to provide large and sustainable quantities. However, the urine proteome is subject to variability and dilution, necessitating careful consideration of normalization procedures during data analysis.^42^ Numerous studies have demonstrated that urine can serve as a valuable source of early biomarkers for a range of diseases.^43^, ^44^, ^45^, ^46^ Tissue samples obtained from surgery or puncture are an important resource for exploring the molecular mechanisms of disease progression. The homogenization process during protein extraction is highly important, as any incomplete homogenization could lead to the loss of vital information, thereby impacting the accuracy and precision of the findings. Common methods for tissue sample extraction include grinding, homogenization, and ultrasonic treatment, among others.^47^ The fundamental principle revolves around minimizing protein degradation, preventing contamination and conducting the process in a controlled environment with low temperatures and lysate preservation.^48^

In addition to analyzing the proteome of complex clinical samples, deep glycosylation analysis of target glycoproteins through antibody affinity and lectin affinity is crucial for identifying disease biomarkers. For example, we isolated IgG from human plasma via immobilized protein A/G agarose and examined its N‐glycosylation in various chronic kidney diseases (CKDs).^49^ Furthermore, we extracted uromodulin from human urine using diatomaceous earth powder and analyzed its N‐glycosylation in IgA nephropathy (IgAN).^50^ In summary, when selecting a protein extraction method, it is important to consider the experimental objectives and sample types and adhere to the fundamental principles outlined above.

#### 2.3.2 Enzymatic digestion

Enzymatic digestion typically does not necessitate special requirements for the analysis of IGPs from complex clinical samples. Trypsin is the predominant enzyme utilized for digestion in IGP analysis via bottom‐up shotgun proteomics (Figure 2B). However, when specific glycoproteins are analyzed, glycosite prediction is necessary to choose the appropriate enzyme or combination of enzymes. For example, the recombinant severe acute respiratory syndrome coronavirus‐2 (SARS‐CoV‐2) spike protein contains 22 potential N‐glycosites. Theoretical analysis of the enzymatic sites revealed that trypsin alone did not generate peptides of sufficient length to cover all potential N‐glycosites. To address this issue, the endoproteinase Glu‐C was introduced to identify the missing N‐glycosites.^51^ Similarly, Watanabe et al.^52^ digested the spike protein separately with trypsin, chymotrypsin, or alpha lytic protease to cover all potential N‐glycosites.

Peptide N‐glycosidase F (PNGase F) is an enzyme derived from *Elizabethkingia miricola* that is produced through *Escherichia coli* recombinant technology. It can effectively remove N‐linked oligosaccharides by cleaving the innermost N‐acetylglucosamine (GlcNAc) and asparagine residues at the junction of almost all types of N‐linked polysaccharides, including high mannose, hybrid, and complex glycans.^53^ Therefore, PNGase F is commonly utilized for the identification of N‐glycans and N‐glycosites.^54^ Additionally, when O‐glycosylation analysis is needed, PNGase F is employed to eliminate N‐glycans to minimize interference with the detection of O‐glycans.^55^ The lack of a definitive protein consensus sequence for O‐glycosites poses a challenge for their identification. However, recently, a versatile O‐glycoprotease known as immunomodulating metalloprotease from *Pseudomonas aeruginosa* was discovered. This enzyme can specifically recognize and cleave glycoproteins adjacent to O‐glycosylated serine or threonine residues, facilitating the accurate identification of O‐glycan structures at each O‐glycosite.^56^, ^57^, ^58^, ^59^, ^60^, ^61^ Using this innovative enzyme, researchers have identified nearly 100 O‐glycoproteins in mouse brains.^57^ In addition to the specific proteases mentioned above, nonspecific proteases can also be utilized to detect specific glycosylation. For example, the use of protease K as a nonspecific serine protease is valuable for overall protein digestion. Employing various enzymes and their combinations will enhance the comprehensive identification of glycosites and glycans.^62^

#### 2.3.3 IGP enrichment

In clinical samples, the low abundance of glycoproteins and IGPs, along with the ion suppression of native IGPs during MS analysis and complex site‐specific heterogeneity, have posed challenges for accurate detection.^63^ To address these challenges, various enrichment methodologies have been developed to increase the detectability and sensitivity of IGPs during MS analysis (Figure 2B). These methodologies include the utilization of specific lectins for capture, hydrazide‐based capture techniques, and affinity separation strategies.^64^ Among the most prevalently employed techniques for glycoprotein or IGP enrichment are lectin affinity chromatography (LAC), boronic acid chemistry, hydrophilic interaction liquid chromatography (HILIC), and hydrazide chemistry. These methods have undergone rapid advancements to improve the enrichment efficiency for glycoproteins or IGPs.^64^, ^65^ A comprehensive overview of the advantages and disadvantages of each enrichment method is shown in Table 1. LAC has been widely used for the study of glycoproteins with special glycans. Lectins, derived from plants or animals, have unique binding sites tailored to recognize specific glycans.^66^ This characteristic has made them invaluable tools in the scientific community for decades, particularly in identifying new disease‐related biomarkers.^67^, ^68^, ^69^, ^70^ The boronic acid chemistry method is known for its reversible reactions with glycans that have cis 1,2 and 1,3 diols, forming cyclic esters under alkaline conditions and releasing the glycans under acidic conditions while maintaining their structure.^71^, ^72^ The HILIC method is emerging as a promising approach for separation and enrichment in glycoproteomics, as it excels at isolating IGPs and glycans. This technique capitalizes on the contrasting electrical properties of ions, hydrophilic IGPs, and relatively hydrophobic nonglycosylated peptides.^73^ This unique configuration enables HILIC to effectively bind IGPs on the chromatographic column, facilitating the removal of nonglycosylated peptides.^74^ The hydrazide chemistry method is employed for the modification of oxidized sugars by hydrazide reagents. It encompasses a series of steps including oxidation, linkage formation, proteolytic hydrolysis, isotope labeling, release, and subsequent analysis.^75^ This highly efficient method provides a robust means for exploring glycosites.^76^, ^77^ The functionalized MNP method has been utilized to enrich glycoproteins, expanding the application range of magnetic nanomaterials. Various nanoparticles act as capsules to bind glycoproteins or IGPs.^78^ For example, a hydrophilic nanomaterial called Fe_3_O_4_@mSiO_2_@G6P has been specifically designed to capture IGPs from both horseradish peroxidase and IgG digests.^79^ Additionally, an ultra‐hydrophilic mesoporous silica magnetic nanosphere, known as Fe_3_O_4_–CG@mSiO_2_, has shown outstanding performance with superior adsorption capacity, sensitivity, size‐exclusion functionality, stability, and recovery efficiency. This makes it highly effective in extracting serum exosomes.^80^ Therefore, it is crucial to carefully choose the suitable enrichment techniques on the basis of specific research requirements, ensuring a balance between sensitivity, specificity, and throughput. Each method offers distinct characteristics that address various aspects of glycoprotein analysis, ranging from the accuracy of glycoprotein capture to the effectiveness of glycopeptide recovery. As the field has advanced, improvements in these techniques are essential for enhancing our comprehension of glycoprotein functions and their significance in diverse biological scenarios.

**TABLE 1:** Brief conclusions of the major enrichment methods.

Enrichment method	Advantages	Disadvantages
Lectin affinity chromatography (LAC)	High specificity, wide variety, commercially available, can recognize glycan, glycoprotein, and glycopeptide^83^, ^84^	High demand for experimental apparatus, deficiency in recognizing specific compositions, and instability^84^, ^85^, ^86^
Boronic acid chemistry	Easy for operation, high structural completeness, permeability, and affinity^87^	Week interaction between boric acid and glycan, highly depends on the pH^87^, ^88^
Hydrophilic interaction liquid chromatography (HILIC)	Skilled in analyzing intricate glycans and detecting various types of samples^89^	Nonspecificity, susceptible to hydrophilic peptide interference^90^
Hydrazide chemistry	Extinguished selectivity, specificity, and reversibility^91^, ^92^	Low capacity, time‐consuming operation, and vulnerable to interference^77^, ^92^, ^93^
Functionalized magnetic nanoparticles (MNPs)	High adsorb capacity, sensitivity, and stability^94^	Low biocompatibility and poisonous for alive cells^95^, ^96^

Enrichment techniques have played a crucial role in advancing glycoproteomics, significantly contributing to the comprehension and analysis of glycoproteins and IGPs. However, it is essential to acknowledge a critical issue: the enrichment process inevitably results in the partial loss of the structural or compositional integrity of glycoproteins or IGPs owing to the preference of each method. This loss can have an impact on the outcomes of glycoproteomic analysis to a certain extent. In response to this challenge, recent advancements have focused on developing combined methods to isolate glycoproteins or IGPs in a more intact or complementary form, with the goal of improving the accuracy and reliability of IGP analysis.^81^, ^82^

### 2.4 LC–MS/MS analysis

Choosing a suitable analysis method (MS instrumentation, fragmentation strategy or MS acquisition method) prior to initiating experimental procedures is crucial for obtaining meaningful outcomes (Figure 2C). Importantly, the resolution and sensitivity of MS instrumentation are pivotal in accurately identifying IGPs. Techniques such as chromatography, which leverages chromatographic columns with varying diameters, play a fundamental role in this endeavor.^97^ The flow rate can be affected by the size of the particles within the column as well as the column's diameter. Generally, using columns with smaller diameters and reducing the flow rate can significantly enhance the separation efficiency of IGPs.^98^, ^99^ The use of MS‐compatible solvents enables the simultaneous initiation of ultra‐performance or high‐performance liquid chromatography (UPLC/HPLC) in tandem with MS. Moreover, the technique of reversed‐phase HPLC (RP‐HPLC) has gained widespread application owing to its compatibility with electrospray ionization.^74^ This compatibility not only facilitates superior separation capabilities but also ensures the production of consistent and reproducible results. The distinct advantages of RP‐HPLC, including its exceptional separation efficiency and reliability, underscore its critical role in the field of analytical chemistry.^100^, ^101^

The journey of MS development is both intricate and fascinating, marking significant milestones in the realm of scientific instrumentation.^102^ Among the various techniques that have emerged, matrix‐assisted laser desorption ionization (MALDI) mass spectrometry stands out for its ability to analyze glycan compositions. This technique is celebrated for its high throughput and efficiency, making it a cornerstone in the field of glycomics.^103^ On the other hand, LC–MS/MS has more advantages in detecting IGPs with high resolution and richer glycosylation information.^10^ This sophisticated MS analysis process unfolds through three pivotal steps: ionization (transforming substances into ions), mass analysis (mass–charge ratio (*m*/*z*)), and detection (mass analyzer and detector), each of which plays a crucial role in the journey from sample to insight. In recent advancements, time‐of‐flight (TOF) and (Orbitrap) mass spectrometers have emerged as frontrunners for clinical glycoproteomic analysis. These technologies offer enhanced sensitivity and precision, making them the best choice for unraveling the complex protein and glycan compositions of clinical samples.^102^

Recent advancements in the field of clinical glycoproteomics have led to the emergence of various tandem MS/MS fragmentation techniques and MS acquisition methods (such as data‐independent acquisition [DIA], data‐dependent acquisition [DDA], etc.), each offering unique insights into the complex world of glycoproteins, owing to the varying spectral information and complexity they yield. Among these methods, stepped collision energy/higher‐energy collisional dissociation (sceHCD), electron‐transfer/higher‐energy collisional dissociation (EThcD), and the combined approach of EThcD and sceHCD (EThcD–sceHCD) represent particularly valuable approaches for clinical glycoproteomics.^104^ sceHCD, the most frequently used technology in N‐glycoproteomics, is capable of producing a rich array of fragment ions from both the glycan and peptide components of an intact N‐glycopeptide within a single spectral analysis. However, its capacity is limited regarding providing the clear spectral evidence needed to identify the precise locations of N‐glycosites and the specific compositions of N‐glycans when multiple N‐glycosites are present within a single sequence. Moreover, sceHCD is not the go‐to method for analyzing O‐glycoproteomics.^32^ In contrast, EThcD has emerged as a more effective approach for the fragmentation of intact O‐glycopeptides because its ability to generate glycan‐attached *c*/*z* ions not only facilitates the identification of peptides but also aids in deducing the locations of glycosites and their glycan compositions.^105^, ^106^ This cutting‐edge approach significantly increases the precision in identifying O‐glycosites, as demonstrated by the increased diversity and number of fragment ions detected. Despite these technological advancements, challenges remain in enhancing the efficiency of EThcD for broader applications in glycoproteomics. In our recent study, we introduced an innovative hybrid dissociation approach, EThcD–sceHCD, which combines the strengths of EThcD and sceHCD. This integration forms a powerful tool within a glycoproteomic workflow, capitalizing on the unique benefits of both techniques.^49^, ^107^ Our findings demonstrate that EThcD–sceHCD significantly enhances the analysis of complex glycoproteins from clinical samples, such as the highly glycosylated human immunodeficiency virus (HIV)‐1 gp120 protein and Igs.^49^, ^108^ Compared with traditional methods that rely solely on EThcD or sceHCD, this hybrid approach has shown superior performance. It delivers higher‐quality spectral data, yields more detailed fragment ion information, and identifies a larger number of intact N/O‐glycopeptides.^32^ These advancements underscore the potential of EThcD–sceHCD to push the boundaries of glycoproteomic research, offering a more thorough and insightful analysis of glycoproteins in clinical samples.^104^

The diverse range of fragmentation techniques plays a pivotal role in propelling forward our comprehension of the functional glycoproteins that are involved in health and disease. This variety enables researchers to unearth in‐depth insights that a uniform method cannot offer. As we delve deeper into the capabilities of these MS/MS techniques, their significant contributions to clinical glycoproteomics are anticipated to enrich our understanding and pave the way for novel research and clinical applications. Nonetheless, the processing of data, especially the annotation of intact N/O‐glycopeptide MS/MS data, presents a formidable challenge. This includes accurately identifying glycan compositions and structures, glycosites, and the peptide backbone.^33^ Therefore, the development of specialized software and bioinformatic tools is highly important.

### 2.5 Bioinformatics analysis

Precise analysis software is dedicated to deciphering MS data, leveraging serious advanced algorithms. These algorithms are specifically crafted to analyze the distinct characteristics of IGPs, with a focus on improving computational speed and efficiency. Currently, these software tools focus primarily on analyzing intact N/O‐glycopeptides, which are closely associated with the development and progression of different diseases.^109^, ^110^ On the basis of variations in core algorithms, these software tools are primarily categorized into peptide‐first searching (such as Byonic, MSFraggerGlyco, etc.) and glycan‐first searching (pGlyco series) (Figure 2D). A comprehensive review has been conducted on their progress, principles, and distinctive features.^35^ Byonic is widely recognized as a commercial software for analyzing MS data from IGPs. Equipped with N‐linked and O‐linked glycan libraries and the targeted protein data bank, this software can automatically search for identifying glycoproteins, glycosites, and glycan compositions.^111^ This software has been effectively utilized to analyze the distinct N‐glycosylation patterns of individual glycoproteins expressed in HeLa cells, as well as the O‐glycosylation profiles of the urinary glycoproteome.^112^, ^113^ MSFragger‐Glyco introduced the innovative approach of open and mass offset search strategies for the initial time. This software is capable of evaluating the energy in mixed glycoproteins by distinguishing the energy levels of glycopeptide fragments in complex situations. Consequently, it can analyze targeted glycopeptides (with higher energy) by disregarding glycan fragments (with lower energy), leading to fewer errors. MSFragger‐Glyco has demonstrated remarkable proficiency in deciphering a multitude of intricate N‐glycoproteome and O‐glycoproteome to date.^114^, ^115^ O‐Pair search is the first algorithm designed for analyzing O‐glycosylation data. This method originally used an ion‐indexed open search with HCD spectra to identify the pairing of peptides and O‐glycan masses rapidly.^61^ The graph‐theoretical approach defines site‐specific O‐glycan localizations on the basis of ions present in EThcD spectra, followed by localization probability calculations via an extension of the phosphoRS algorithm. This algorithm is primarily employed for localizing phosphorylation sites, determining the false discovery rate (FDR), and identifying nonmodified peptides.^61^, ^116^ Glyco‐Peptide Finder (GP Finder) was specifically developed to identify extensive glycosites. By analyzing the data of N‐ and O‐linked glycopeptides resulting from nonspecific proteolysis, the overall probability of peptide sequences after digestion can be calculated while maintaining a 5% FDR. This tool is capable of analyzing not only a single protein with a single glycosite but also an unknown protein mixture.^117^ StrucGP specializes in identifying intact N‐glycopeptides. It begins by identifying the peptide components via the Y‐ion pattern of the N‐glycans and then strives to interpret glycan structures via predefined substructure templates. Additionally, it includes glycan‐level quality control measures after glycan identification.^118^ pGlyco series software, as the first search engine capable of performing quality control at the glycan, peptide, and IGP levels, has significantly enhanced accuracy and expedited IGP matching.^119^ The software tools mentioned above have greatly increased the precision of identifying intact N‐glycopeptides and O‐glycopeptides. Nevertheless, there is still a gap in software tools for IGP quantitative analysis. pGlycoQuant has made significant strides in IGP matching by using a deep learning model to minimize missing values.^120^ PANDA software employs a polynomial expansion technique to compute the natural distribution of peptide elements, thereby deriving the theoretical relative abundance of the isotopic peaks. The observed isotopic intensities were subsequently extracted from the MS1 spectra by matching the theoretical position of an isotopic peak within a user‐defined error tolerance.^121^

The advancements mentioned above have significantly enhanced the quantitative capabilities of popular search engines such as pGlyco3, Byonic, and MSFragger‐Glyco. However, these software tools still face numerous challenges that require resolution. The ideal software should be compatible with various MS acquisition methods, different fragmentation modes, N‐ and O‐glycan libraries, and protein libraries from diverse species. It is essential to develop glycan database‐independent tools to decode new glycans effectively. The creation of software capable of conducting qualitative and quantitative analyses of IGP from multiple sources simultaneously will greatly enhance the clinical application and transformation of clinical glycoproteomics. Overall, current software tools are progressing toward achieving more comprehensive IGP analysis with enhanced accuracy and flexibility.

### 2.6 Result verification

Mass spectroscopy‐based glycoproteomics has enabled the discovery of thousands of differentially expressed glycoproteins, IGPs, glycosites, and glycans in disease and control samples. However, few candidate glyco‐biomarkers have been tested in clinical studies. This phenomenon can be attributed to immature glycoproteomic methodology, a lack of quality control, false positive detection, a small clinical sample size, a lack of quantification and validation experiments, and challenges in validation and clinical application.^122^ Hence, researchers should gradually begin to validate clinical glycoproteomic findings through necessary results verification experiments, similar to clinical proteomics, despite its challenges.^36^ As shown in Figure 2E, the verification experiments consisted of molecular, cell, and animal experiments. Through these experiments, the structure and function of proteins and glycans can be understood in detail. Furthermore, the validation of glycoprotein biomarkers should be conducted both retrospectively and prospectively, employing multicenter independent sample validation methods. The lack of bias in the verification results is the key to the final evaluation of glycoprotein biomarker performance.^123^

In general, the methodology of clinical glycoproteomics involves several procedures (clinical sample selection, sample processing, LC–MS/MS analysis, bioinformatics analysis, and result verification) mentioned above. These methodologies are continually refined to improve the quality of results, leading to a deeper understanding of the pathophysiology of diseases at the glycoproteomic level. However, it is important to note that these methodologies still face challenges, such as the inadvertent selection of inappropriate samples, manual experimental processes, inappropriate MS methods, limited bioinformatic tools, and uncertain verification results.^124^ Importantly, only high‐quality samples, standardized sample processing, along with reliable measurements and analysis, are valuable for ensuring the reproducibility and reliability of experiments. By addressing these obstacles, clinical glycoproteomics can continue to make significant contributions to our understanding of diseases.^33^, ^125^

## 3 CLINICAL GLYCOPROTEOMICS AND HUMAN DISEASES

The advancement in clinical glycoproteomic methods has significantly enhanced their application across a broad spectrum of diseases. This includes brain diseases, CVDs, cancers, kidney diseases, and metabolic diseases. These innovations offer promising avenues for understanding disease mechanisms, improving diagnostics, and tailoring personalized treatment strategies. Herein, we aim to comprehensively review and discuss the role of glycoproteins in disease mechanisms, highlighting their importance in both diagnosis and therapeutic interventions. By doing so, we aspire to offer a clearer direction for future clinical glycoproteomics research, potentially paving the way for groundbreaking discoveries in disease management and treatment strategies.

### 3.1 Brain diseases

#### 3.1.1 Alzheimer's disease

**FIGURE 3:**
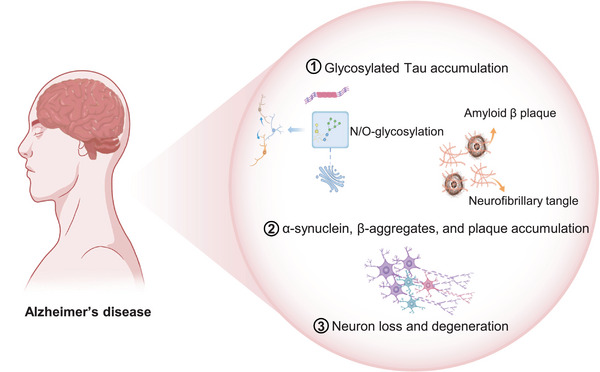
Pathogenesis of Alzheimer's disease. This diagram illustrates the three primary stages involved in the development of Alzheimer's disease (AD). Initially, the tau proteins undergo glycosylation within the Golgi apparatus, leading to their accumulation in neurons. This process is followed by the production of amyloid β plaques and the formation of neurofibrillary tangles. These pathological alterations ultimately result in neuronal loss and degeneration, contributing to the cognitive impairment associated with AD. Tau serves as a key microtubule‐associated glycoprotein in mature neurons (by Biorender).

According to epidemiological data, the global population at risk for Alzheimer's disease (AD) is projected to double by 2050.^126^ There are three primary stages involved in the development of AD (Figure 3). Initially, the aggregated tubulin‐associated unit (Tau) proteins undergo glycosylation within the Golgi apparatus, leading to their accumulation in neurons. This process is followed by the production of amyloid β plaques and the formation of neurofibrillary tangles. These pathological alterations ultimately result in neuronal loss and degeneration, contributing to the cognitive impairment associated with AD. The accumulation of amyloid β protein, primarily caused by genetic variants such as apolipoprotein E (APOE), along with the presence of neurofibrillary tangles triggered by Tau, represents significant pathophysiological alterations associated with AD.^127^, ^128^, ^129^ Recent advancements in clinical glycoproteomic techniques have revealed that these complex glycoproteomic interactions are involved in the pathogenesis of AD. For instance, Zhang et al.^130^ identified six N‐glycoproteins associated with AD phenotypes via ZIC–HILIC and LC–MS/MS techniques. These glycoproteins influence the functions of the extracellular matrix, synapses, and organelles, as well as neuroinflammation, cell adhesion, and endocytic trafficking. In the same year, the N‐glycoproteomic landscape in the brains of AD (APP/PS1 transgenic) mice revealed dysregulation of glutamate receptors, as well as fucosylated and oligomannose glycans.^131^ Suttapitugsakul et al.^132^ profiled N‐glycoproteins in normal, asymptomatic, and symptomatic AD brains from humans. Their findings revealed that the brain predominantly contains high‐mannose, fucosylated, and bisected N‐glycans, with distinct expression patterns observed across different stages of AD through the use of multilectin enrichment, HILIC, and LC–MS/MS techniques. These studies revealed that N‐glycosylation plays a multifaceted role in the pathogenesis of AD, offering valuable insights into the interpretation of circulating glycoproteins present in the CSF of AD patients and their potential diagnostic value. Furthermore, Losev et al.^133^ conducted a thorough investigation of N‐glycosylation in wild‐type human tau. Their analysis revealed that the asparagine residues at positions N359 and N410 were N‐glycosylated. However, this N‐glycosylation was absent in mutants in which asparagine was replaced with glutamine at positions N167Q, N359Q, and N410Q. Furthermore, a patient with an intermediate bisecting GlcNAc‐to‐tau ratio in CSF was found to have an elevated risk of AD, with a hazard ratio of 2.06 and a 95% confidence interval (CI) of 1.18‐3.6.^134^, ^135^ Similarly, another study identified 32 N‐glycosites that were modified with bisecting GlcNAc.^136^ This modification, catalyzed by acetylglucosaminyltransferase‐III, involves the transfer of GlcNAc to the mannose of an N‐glycan through a β‐1,4‐linkage, which exacerbates the onset of AD. In contrast, dihydroergocristine mesylate has been shown to alleviate spatial memory disorders and Alzheimer's‐type changes by mitigating aberrant bisecting N‐glycosylation.^137^ Neuroinflammation is widely recognized as a central component in the pathogenesis of AD. Consequently, immunological glycoproteins may serve as underlying biomarkers for the precise detection of AD. For instance, autoantibodies are naturally produced to defend against amyloid beta. A recent study analyzed the glycan structure of AD autoantibodies via MALDI–TOF–MS.^138^ The results indicated that N‐glycosylation plays an important role in aggregation, toxicity, and phagocytosis, while the specificity and sensitivity for predicting the disease state could reach 100 and 95%, respectively.

In parallel, O‐glycosylation also influences the onset of AD. Previous studies have demonstrated that amyloid β, a fragment of amyloid precursor protein (APP), is a vital component of pathological senile plaques.^139^ However, O‐glycosylation at the cleavage site affects the synthesis and production of amyloid proteins, which undoubtedly impacts the progression of AD.^140^ In 2022, a comprehensive platform was developed that combines universal boronic acid enrichment, high‐pH fractionation, and EThcD methods to provide an unbiased profile of O‐glycosylation in both AD patients and healthy individuals.^141^ A total of 308 O‐glycopeptides, encompassing both sialylated and nonsialylated forms, were identified. Additionally, a decreasing trend in fucosylation, along with an increase in endogenous O‐glycosylation, may contribute to the progression of AD.^141^ Additionally, Shi et al.^142^ developed a targeted MS approach utilizing multifragmentation techniques for the identification of O‐glycosites. A total of 14 O‐glycosites were identified on APP, with at least four of these O‐glycosites containing N‐acetyl galactosamine (GalNAc) (Tn antigen), core 1, and mono‐ or di‐sialylated cores. The hyper‐O‐glycosylated form of APP is more susceptible to degradation into amyloid β.^143^ In neurons affected by AD, hypo‐O‐glycosylated APP is transported back to the Golgi apparatus, where it acquires additional O‐glycans.^143^ This untypical O‐glycosylation pathway allows hyper‐O‐glycosylated APP to contribute to the pathological production of amyloid β, indicating its potential as a target for diagnosis and therapy. Krüger et al.^144^ initially reported a method for differentiating AD through the analysis of free glycans in CSF. These findings indicated that sialylated glycosylation was more prevalent than O‐glycosylation when it was completely occupied by N‐ and O‐glycans or monosaccharides. They also demonstrated the ability of some glycans to effectively discriminate between AD patients and healthy controls. Furthermore, a novel category of compounds capable of inhibiting de‐O‐glycosylation was identified, showing potential for alleviating AD symptoms.^145^ By targeting O‐GlcNAcase (OGA), a critical enzyme responsible for the dissolution of O‐glycans, the activities of human OGA and HexB are suppressed. This suppression holds promise for the development of effective anti‐tau phosphorylation agents for the treatment of AD.

#### 3.1.2 Other neurological diseases

Other neurological diseases, including neurodegenerative disorders, neuropsychiatric disorders, and neurodevelopmental diseases, are influenced to varying degrees by glycoproteins. Owing to the proliferation of novel glycoproteomic techniques, researchers have begun to explore these influences more thoroughly. Like AD, Parkinson's disease (PD) is a neurodegenerative disease that is affected by glycoproteins; however, there is limited evidence to support this relationship. A recent N‐glycoproteomic study revealed an increase in core fucose, sialic acid, and bisecting GlcNAc at the overall glycan level and specific glycosites of proteins associated with PD.^68^ In the context of neuropsychiatric disorders, abnormal N‐glycosylation of γ‐aminobutyric acid receptors (α1, β1, and β2) has been investigated in the brains of individuals with schizophrenia, indicating that changes in N‐glycosylation may contribute to atypical neurological emotions.^146^ A recent study revealed that missense variants in the solute carrier family 39 member 8 (SLC39A8) gene are strongly associated with N‐glycosylation.^147^ Homozygous loss‐of‐function mutations in SLC39A8 lead to decreased plasma manganese (Mn) levels. Importantly, since Mn plays a crucial role in N‐glycan biosynthesis, analyses of N‐glycosylation have shown an increase in precursor N‐glycans alongside a decrease in complex N‐glycans.^147^ Additionally, genome‐wide association studies (GWASs) have demonstrated that genes involved in O‐glycosylation contribute to the pathogenesis of schizophrenia.^148^, ^149^ For example, polypeptide‐N‐acetylgalactosaminyltransferase 10 can influence glycosylation enzymes in the brain and has been identified as having a strong link to schizophrenia.^149^ When glycosylation is associated with neurodevelopmental disorders, related discoveries are notably rare. A recent study revealed that abnormal N‐glycosylation of X chromosome‐linked neuroligin 4 (NLGN4) is associated with the development of autism spectrum disorder. A mutation in NLGN4 disrupts N‐glycosylation at the adjacent N102 glycosite, impairing surface trafficking and normal synaptic function.^150^ Rett syndrome, considered a subset of autism spectrum disorder, is primarily attributed to mutations in the methyl‐CpG binding protein 2 (MECP2) gene.^151^

Specifically, defects in O‐GlcNAcylation at threonine 203 within mutations of the MECP2 gene have been found to interfere with synaptic transmission in both cultured cells and the cortex of mouse models, leading to various dysfunctions.^152^ Indeed, increasing evidence has demonstrated a link between abnormal glycosylation and brain diseases. However, the precise relationships between specific glycosylation patterns and individual diseases remain unclear. The factors influencing thoughts, behavior, and development are complex and demand further investigation.

### 3.2 Cardiovascular diseases

**FIGURE 4:**
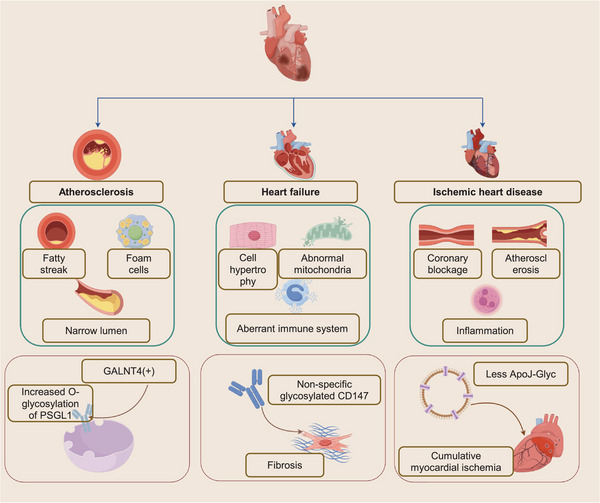
Pathogenesis of abnormal glycosylation and external pathological manifestations associated with atherosclerosis, heart failure, and ischemic heart disease. AS is associated with GALNT4, which modifies the O‐glycosylation of PSGL‐1, thereby contributing to the progression of AS. Heart failure is linked to myocardial fibrosis, along with dysregulated glycosylated CD147, which impacts both cell hypertrophy and the immune response. Additionally, reduced levels of ApoJ–Glyc are induced during myocardial ischemia (by Figdraw). AS, atherosclerosis; HF, heart failure; IHD, ischemic heart disease; GALNT4, polypeptide N‐acetylgalactosaminyltransferase 4; PSGL1, P‐selectin glycoprotein ligand 1; CD147, extracellular matrix metalloproteinase inducer; ApoJ–Glyc, glycosylated apolipoprotein J.

Glycoproteins essentially influence the formation and regulation of myocardial cells and vascular endothelial cells, enabling them to carry out physiological functions, maintain metabolism, and maintain homeostasis.^153^ Conversely, abnormal glycosylation can lead to the development of CVDs. Current research has focused primarily on identifying aberrant glycosylation processes and patterns, aiming to elucidate the internal link between abnormal glycosylation and external pathological manifestations, particularly in the context of atherosclerosis (AS), heart failure (HF), and ischemic heart disease (IHD), which will be discussed in detail below (Figure 4).^154^, ^155^, ^156^ Finally, we summarize and discuss the advancements made in glycoproteomic research on CVDs, along with the existing limitations.

#### 3.2.1 Atherosclerosis

The traditional mechanism of AS comprises three primary stages, the formation of fatty streaks, and the development of atheroma and atherosclerotic plaques, all of which are accompanied by inflammation (Figure 4). The accumulation of oxidized low‐density lipoprotein (OxLDL), macrophage (especially foam cells), smooth muscle cells (SMCs), and cell debris in the intimal space of vascular endothelial cells, will lead to vascular lumen stenosis, even vascular occlusion and myocardial infarction (MI).^157^ Currently, as low‐density lipoprotein (LDL) levels, smoking rates, and blood pressure have decreased, the profiles of risk factors have shifted to some extent, accompanied by an increase in younger AS patients. This novel finding also connects the inflammatory pathway and the abnormal appearance of white blood cells with the progression of AS.^158^ With the advancement of biomarker detection technology, there is a growing focus not only on LDL but also on immune processes and cell interactions.^159^ By elucidating the mechanism underlying AS clearly and systematically, precise diagnosis and therapeutics are becoming feasible in the future. Glycans and glycan‐binding proteins play crucial roles in disease development. Endothelial proteoglycans, along with cell‐bound glycoproteins and glycolipids, are barriers that prevent plasma components from interacting with and damaging the integrity of the endothelium, as well as reservoirs for GAG‐binding proteins.^160^ In addition, AS is significantly influenced by hyperlipidemia, particularly elevated levels of triglycerides and cholesterol, which are closely associated with apolipoproteins and lipoproteins.^161^, ^162^ Thus, the glycosylation of these proteins can affect the progression of AS. Notably, a majority of high‐density lipoprotein (HDL) and LDL‐associated proteins undergo glycosylation.^163^ In a comparative study analyzing blood lipid profiles and glycosylated lipoproteins in patients with MI attributed to coronary AS versus healthy individuals, researchers reported a marked increase in the levels of glycosylated hemoglobin (G‐HbA1c), glycosylated HDL (G‐HDL), and glycosylated LDL (G‐LDL) in the serum of patients. Furthermore, the levels of G‐LDL were found to be significantly correlated with glucose levels and HbA1c.^164^ As mentioned previously, inflammation is crucial to the progression of AS. P‐selectin glycoprotein ligand 1 (PSGL1) functions not only as a transmembrane glycoprotein but also as an important selectin ligand on monocytes.^165^ GWASs have indicated that polypeptide N‐acetylgalactosaminyltransferase 4 (GALNT4) is likely associated with susceptibility to AS. More importantly, the expression of GALNT4 can modify the O‐glycosylation of PSGL1, subsequently activating downstream signaling molecules such as Akt/mTOR and IκBα/NFκB, which enhance the adhesion and migration of monocytes.^166^ This glycosylation process plays a vital role in monocyte recruitment, as well as in the function of adhesion molecules and foam cell membrane proteins, all of which contribute to the progression of AS.^167^ Recent findings have highlighted the pivotal role of various glycoproteins during the disease's progression. Glycoprotein VI (GPVI) has been identified as a crucial marker in evaluating the coagulation level associated with the early stages of AS, such as platelet adhesion. The presence of a higher quantity of GPVI is indicative of an increased likelihood of platelet activation.^168^, ^169^, ^170^ This correlation underscores the significance of GPVI in the early detection and management of AS. A foundational study demonstrated that inhibiting GPVI, through the administration of anti‐GPVI antibodies, could potentially slow down or halt the progression of AS.^171^ Additionally, an intriguing discovery was made regarding β2 glycoprotein I (β2GPI), extracted from human atherosclerotic plaques. The study shed light on the immune response within the plaques, revealing a predominant infiltration of CD4(+) lymphocytes in areas rich in β2GPI, while CD8(+) lymphocytes were notably absent.^172^ This differential presence of lymphocyte subtypes in response to β2GPI highlights the complex interplay between the immune system and the development of atherosclerotic plaques.

In conclusion, the formation of OxLDL and the activation of macrophages are significant factors in the development of AS. Glycosylations are extensively involved in this process, impacting the structure and function of glycoproteins and contributing to pathological changes.^173^ Therefore, elucidating the abnormalities in the glycosylation pattern during the development of AS not only enhances our understanding of the underlying mechanisms but also offers potential novel therapeutic targets for patients.

#### 3.2.2 Heart failure

HF has emerged as a significant epidemic disease worldwide, and is characterized by high mortality rates, particularly among the aging population.^174^, ^175^ As a progressing disease, HF primarily involves myocardial injury and abnormal contractility of the myocardium.^176^, ^177^ The pathological and physiological alterations associated with HF can be attributed to changes in neurohormonal factors, such as β‐adrenergic hormones, angiotensin, and endothelin, as well as remodeling of the left ventricular myocardium.^178^ Recent clinical studies have revealed that the use of sodium‐glucose cotransporter 2 (SGLT2) inhibitors in the treatment of diabetes not only lowers blood glucose levels but also reduces the incidence of CVDs. Research has demonstrated that SGLT2 plays a crucial role in regulating ketones, carbohydrates, and fatty acids, which are vital for myocardial metabolism, including ion homeostasis and cellular redox reactions.^179^, ^180^ Additionally, some researchers suggest that the onset of HF may be linked to deficiencies in micronutrients that are essential for mitochondrial metabolism, such as iron, selenium and zinc.^181^

Basigin (CD147), also known as extracellular matrix metalloproteinase (MMP) inducer, is a transmembrane glycoprotein that plays a crucial role in the synthesis of MMPs. These enzymes are integral to various biochemical processes, including the remodeling of myocardial tissue.^182^ A recent study revealed that glycosylated CD147 is the major form in heart tissue and is significantly upregulated in response to transverse aortic constriction (TAC) in murine models.^156^ Additionally, the administration of adeno‐associated virus 9 to induce cardiac‐specific overexpression of either wild‐type CD147 or a mutant form of CD147 with three altered glycosites revealed significant insights. Mice that received injections of wild‐type CD147 exhibited marked deterioration in cardiac function following TAC, which affected both myocardial contraction and relaxation. Pathological examination indicated the presence of myocardial hypertrophy, characterized by increased cross‐sectional areas of cardiomyocytes and myocardial fibrosis, alongside elevated levels of hypertrophic markers such as atrial natriuretic factor and brain natriuretic peptide. In contrast, mice expressing specifically glycosylated CD147 demonstrated improved cardiac function, suggesting that glycosylation mitigated the detrimental effects associated with CD147 overexpression. Furthermore, a motif analysis of CD147 presented a potential binding site for tumor necrosis factor receptor‐associated factor 2 (TRAF2), which was previously linked to cardiac dysfunction. Immunoprecipitation studies indicated that wild‐type CD147 interacts with TRAF2, whereas glycosylated CD147 has a significantly reduced binding affinity for TRAF2. Consequently, glycosylated CD147 offers a novel perspective for the treatment of HF.^156^ In 2022, a drug known as stachytine hydrochloride was utilized to inhibit α−1,6‐fucosylation in the N‐glycosylation of the β1 adrenergic receptor (β1AR) in a study conducted in mice. The activation of β1AR facilitates its binding to G‐proteins, which subsequently enhances the synthesis of cyclic adenosine monophosphate (cAMP). This process activates protein kinase A (PKA) and its downstream signaling molecules, ultimately leading to an increase in myocardial contractility.^183^ The LCA method verified that stachytine hydrochloride effectively suppresses the synthesis of α‐1,6‐fucosylation by reducing the activity of α‐1,6‐fucosyltransferase (FUT8) and α‐1,3‐mannosyl‐glycoprotein 4‐β‐N‐acetylglucosaminyltransferase A, while increasing N‐glycosylation on β1AR and sustaining the activation of the cAMP/PKA signaling pathway to protect myocardial cells.^184^ Additionally, MS was employed to investigate the unusual abundance of glycoproteins in extracted serum and tissues from mice with HF. The findings revealed that mice were able to synthesize N‐acetylneuraminic acid (NeuAc). However, this compound is predominantly present in the serum, which aligns with the mechanism of TAC.^185^

In general, glycoproteomics discoveries in HF have significantly enhanced our understanding of the mechanisms underlying this disease (Figure 4). Importantly, clarifying the abundance and quality of critical glycoproteins, as well as establishing standardized comparisons between HF patients and healthy controls, is crucial for effective prevention, diagnosis, and treatment. Current research tends to focus on linking abnormally glycosylated inflammatory factors with the mechanisms of HF.^186^ Future studies should explore anti‐inflammatory strategies and the regulation of cardiomyocyte metabolism to potentially slow or even reverse cardiac remodeling.

#### 3.2.3 Ischemic heart disease

The incidence rate of IHD is increasing globally, leading to increased occurrence of MI, which is associated with high mortality rates, particularly among women.^187^ Researchers believe that the fundamental mechanism underlying IHD involves a dynamic process of AS or alterations in coronary circulation that result in diminished cardiac blood flow, ultimately leading to insufficient myocardial oxygen supply.^188^, ^189^ Throughout this dynamic process, various biomarkers, including cardiac troponin, microRNA, and systemic inflammatory index, are released from cells into the bloodstream.^190^, ^191^ These biomarkers are instrumental in the prevention and diagnosis of IHD. Below, we elaborate on certain glycoprotein‐related biomarkers.

A multicenter cohort study revealed that apolipoprotein J (ApoJ), a cytoprotective and antioxidant glycoprotein, is significantly induced during myocardial ischemia, and concurrently shows reduced levels of glycosylated ApoJ (ApoJ–Glyc). Following the percutaneous intervention, an increase in ApoJ–Glyc levels was observed, suggesting that ApoJ–Glyc may serve as a novel biomarker for early diagnosis.^192^ While it is essential to comprehensively discuss the alterations associated with IHD, it is equally important to focus on the outcomes of IHD, such as acute myocardial infarction (AMI) and myocardial ischemia–reperfusion (I/R) injury. A study analyzing the specific N‐glycosylation and O‐glycosylation of apolipoprotein A‐I (Apo A‐I) in AMI revealed that changes in the 45‐kDa Apo A‐I were linked to N‐glycosylation, whereas alterations in the 28‐kDa Apo A‐I were associated with O‐glycosylation.^193^ During the process of myocardial I/R injury, alterations in myocardial mitochondrial metabolism‐related glycoproteins occur. A study utilizing LC–MS/MS and bioinformatics analysis established a rat model of myocardial I/R injury and revealed that various glycoproteins, including GalNAcα/β1‐3/6 Gal, GalNAcα1,3 Gal, and GalNAcα‐1 and 3Galβ‐1, were significantly different from those in the sham‐operated group. Notably, the number of Siaα2‐6 Gal/GalNAc structures, which are recognized by sambucus nigra lectin (SNA), is significantly increased.^194^ Compared with those related to AS and HF, glycoproteomic discoveries related to IHD remain insufficient for clinical diagnosis. One contributing factor is that IHD is often diagnosed concurrently with AMI, which heavily depends on clinical performance and electrocardiogram results.^195^, ^196^ However, the occurrence of AMI is strongly associated with high mortality. Therefore, identifying IHD‐associated biomarkers and facilitating early diagnosis and prevention are crucial steps in mitigating the risk of AMI.

Conclusively, extensive research indicates that glycoproteins play crucial roles in regulating cardiovascular system functions, whereas abnormal glycosylation can lead to cardiovascular disorders. Although we emphasize the importance of glycosylation in CVDs, it is essential to recognize that our current understanding remains limited, particularly regarding the detailed mechanisms underlying CVDs.^197^, ^198^ Moreover, drawing from insights gained in the fields of cancer and autoimmune diseases, contemporary research tends to focus on identifying inflammatory factors associated with CVDs and their aberrant glycosylation patterns.^199^, ^200^, ^201^ Given the diverse types of cells involved in CVDs, employing single‐cell techniques to investigate protein glycosylation could yield substantial benefits.^202^, ^203^ Finally, exploring cell interactions is crucial for gaining a deeper understanding of the occurrence and progression of CVDs. However, current research has focused primarily on elucidating the changes among different types of vascular endothelial cells, rather than systematically describing the interactions between cells involved in the metabolism of the circulatory system and linking these interactions to specific pathophysiological changes.^204^, ^205^ In the future, advancements in glycoproteomics are expected to yield new discoveries that will significantly enhance the clinical prevention, diagnosis, and treatment of CVDs.

### 3.3 Cancers

**FIGURE 5:**
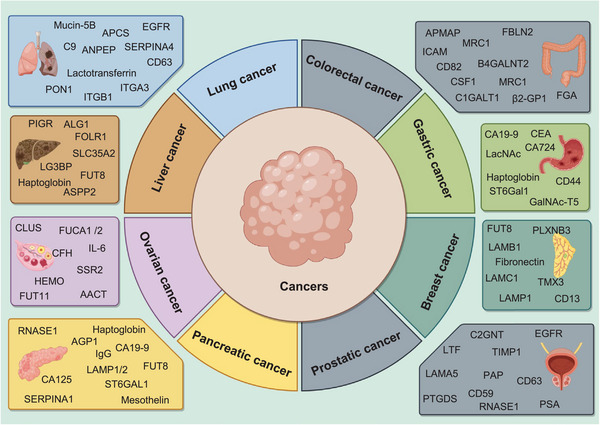
Primary glycoprotein biomarkers associated with cancers include abnormal glycoproteins and glycoprotein‐related enzymes. The glycoproteic biomarkers of lung cancer include epidermal growth factor receptor (EGFR), amyloid p component serum (APCS), complement component 9 (C9), kallistatin (SERPINA4), paraoxonase/arylesterase 1 (PON1), alanyl aminopeptidase (ANPEP), integrin beta‐1 (ITGB1), integrin alpha 3 (ITGA3), mucin‐5B, lactotransferrin, and cluster of differentiation 63 (CD63). The biomarkers of colorectal cancer incorporate intercellular adhesion molecule 1 (ICAM1), adipocyte plasma membrane‐associated protein (APMAP), beta‐2‐glycoprotein 1 (β2‐GP1), core 1 β1,3‐galactosyltransferase (C1GALT1), fibroblast growth factor receptor 2 (FGFR2), beta‐1,4‐N‐acetyl galactosaminyl transferase 2 (B4GALNT2), cluster of differentiation 82 (CD82), colony‐stimulating factor 1 (CSF1), macrophage mannose receptor 1 (MRC1), fibrinogen alpha chain (FGA) and fibulin‐2 (FBLN2). The biomarkers of gastric cancer comprise carbohydrate antigen 19‐9 (CA19‐9), carbohydrate antigen 724 (CA724), haptoglobin (Hp), cluster of differentiation 44 (CD44), carcinoembryonic antigen (CEA), β‐galactoside α2,6‐sialyltranferase 1 (ST6Gal1), N‐acetyllactosamine (LacNAc), and N‐acetylgalactosaminyl transferase‐5 (GalNAc‐T5). The biomarkers of breast cancer have α‐1,6‐fucosyltransferase (FUT8), cluster of differentiation 13 (CD13), lysosomal‐associated membrane protein 1 (LAMP1), fibronectin, plexin‐B3 (PLXNB3), laminin β‐1 (LAMB1), laminin subunit gamma 1 (LAMC1), thioredoxin‐related transmembrane protein 3 (TMX3). When it refers to prostatic cancer, epidermal growth factor receptor (EGFR), cluster of differentiation 59 (CD59), cluster of differentiation 63 (CD63), core 2 β6‐GlcNAc‐transferase (C2GnT), tissue inhibitor metalloproteinase 1 (TIMP1), prostate‐specific antigen (PSA), prostatic acid phosphatase (PAP), lactotransferrin (LTF), prostaglandin‐H2 D‐isomerase (PTGDS) and ribonuclease A family member 1 (RNASE1) are considered as promosing biomarkers. In terms of the ovarian cancer, carbohydrate antigen 125 (CA125), fucosidases (FUCA) 1/2, clusterin (CLUS), complement factor H (CFH), hemopexin (HEMO), interleukin‐6 (IL‐6), fucosyltransferase 11 (FUT11), and α1‐antichymotrypsin (ACCT) are the major biomarkers. Pancreatic cancer biomarkers include mesothelin, lysosomal‐associated membrane protein 1/2 (LAMP1/2), α‐1,6‐fucosyltransferase (FUT8), haptoglobin (Hp), immunoglobulin G, carbohydrate antigen 125 (CA125), ribonuclease A family member 1 (RNASE1), ST6 beta‐galactoside alpha‐2,6‐sialyltransferase 1/2 (ST6GAL1/2), α1‐acidglycoprotein (AGP1), and serpin family A member 1 (SERPINA1). The diagnostic and therapeutic targets of liver cancer contain asparagine‐linked glycosylation protein 1 homolog (ALG1), haptoglobin (Hp), α‐1,6‐fucosyltransferase (FUT8), folate receptor alpha (FOLR1), solute carrier family 35 member A2 (SLC35A2), polymeric immunoglobulin receptor (PIGR), apoptosis stimulating of p53 protein 2 (ASPP2), and lectin galectin‐3‐binding protein (LG3BP) (by Figdraw).

Cancer stands as a formidable adversary in global health, with approximately 10 million new cases and over 6 million fatalities annually worldwide. Among the various types of cancers, lung, colorectal, gastric, and breast cancers are notably prevalent.^206^, ^207^, ^208^ The factors contributing to cancer are multifaceted. Advances in biological science have improved our understanding of cancer, highlighting gene mutations, infections, and environmental factors as primary contributors to its development.^209^, ^210^, ^211^ To unravel the mysteries of cancer, researchers have identified several tumor‐derived biomarkers, including genes, proteins, and their modified forms such as glycoproteins, along with metabolic pathways. These discoveries have been pivotal in enhancing the diagnosis and treatment of cancer.^212^, ^213^, ^214^, ^215^ Traditional cancer treatments include surgery, radiotherapy, and chemotherapy. Each of these methods has unique clinical benefits and limitations, often accompanied by various complications.^216^, ^217^, ^218^ Over the past few decades, immunotherapy has emerged as a groundbreaking and innovative treatment, featuring swift advancements and demonstrating remarkable efficacy in combating tumors.^219^, ^220^ This approach has revolutionized the way we approach cancer treatment, highlighting the importance of targeted molecular therapies. These therapies are designed to disrupt the metabolic processes of tumor cells, leading to their death while sparing healthy cells.^221^, ^222^ This precision is achieved through the identification of specific molecular targets, a process greatly facilitated by advancements in omics technologies—including genomics, proteomics, and metabolomics. These technologies enable the detailed analysis of abnormal reactions, enzymes, and products of cellular metabolism, significantly contributing to our understanding of tumor biology and the development of effective anticancer drugs.^223^, ^224^, ^225^, ^226^ Furthermore, as research delves deeper into the relationship between proteins and cancer, the field of glycoproteomics has come to the forefront. Glycoproteins, which are functional proteins, have been identified as key players in the initiation and progression of cancer. The insights gained from studying these glycoproteins have not only enriched our understanding of the disease but also opened new avenues for therapeutic intervention.^227^, ^228^, ^229^, ^230^ Figure 5 shows glycoproteins that can be used as potential diagnostic biomarkers and therapeutic targets in different cancers, such as lung cancer, colorectal cancer (CRC), gastric cancer, breast cancer, prostatic cancer, pancreatic cancer, ovarian cancer, and liver cancer. In the following sections, we explore the significant contributions of glycoproteomic research to our understanding of cancer and its application in the development of novel treatment strategies. This exploration will underscore the transformative impact of precision medicine in oncology, offering hope for more effective and less invasive treatment options for patients worldwide.

#### 3.3.1 Lung cancer

Lung cancer is universally recognized as a critical global health issue, characterized by significant annual rates of morbidity and mortality. It has garnered considerable attention and research efforts.^231^, ^232^, ^233^ From a pathological perspective, lung cancer can be classified into two main types: small cell lung cancer (SCLC) and non‐small cell lung cancer (NSCLC). The current therapeutic strategies for NSCLC have received increasing attention because of the elusive nature of SCLC mechanisms, which hampers drug development efforts.^234^, ^235^, ^236^ Furthermore, it is noteworthy that NSCLC has the potential to morph into SCLC, particularly in patients exhibiting mutations in the epidermal growth factor receptor (EGFR).^237^ Groundbreaking research into genetic mutations and the identification of aberrant proteins have paved the way for the development of molecular targeted therapies. These therapies have demonstrated efficacy in curbing the proliferation of tumor cells, marking a significant advancement in the treatment of lung cancer.^238^, ^239^, ^240^

In the realm of clinical glycoproteomics, various biomarkers have been identified or are currently being investigated. Human saliva has proven to be a valuable resource for clinical diagnostics. A study conducted by Liu et al.^241^ involved analyzing saliva samples from healthy individuals and patients with NSCLC. This research revealed that 154 N‐glycosites and 259 site‐specific glycoforms were dysregulated in the NSCLC group. Notably, some N‐glycosites were found within the same glycoprotein, and the glycans attached to these sites presented varying levels of expression. This is particularly evident in glycoproteins such as haptoglobin (Hp), mucin‐5B, lactotransferrin, and α‐1‐acid glycoprotein 1, all of which are closely linked to NSCLC.^241^ In SCLC, the process of fucosylation is notably prevalent. Ahn et al.^242^ identified four major types of fucosylated proteins: amyloid p component serum, complement component 9 (C9), kallistatin, and paraoxonase/arylesterase 1. These glycoproteins have demonstrated significant diagnostic potential for SCLC. Furthermore, small extracellular vesicles (sEVs), which are secreted by tumor cells, carry a wealth of information that is useful for the diagnosis of different subtypes. A particular study highlighted the N‐glycosylation patterns present on sEVs derived from both SCLC and NSCLC cells, underscoring the complexity and potential of glycoproteins in cancer diagnostics.^243^ By meticulously comparing the content of marker proteins, the study revealed that sEVs from tumor cells exhibit distinct types, each characterized by varying levels of marker proteins. This discovery underscores the qualitative differences among sEVs, with a notable emphasis on the cluster of differentiation 63 (CD63) glycoprotein, which features three potential glycosites. Specifically, the core N‐glycan structures present in different sEVs were analyzed. In the case of sEVs derived from three types of NSCLC cells, a predominance of biantennary and triantennary N‐glycans was observed, with a significant presence of core fucosylation. This finding contrasts sharply with that of sEVs from SCLC, which demonstrated vast heterogeneity in N‐glycan structures, reflecting the diversity of SCLC cell types. Moreover, the study leveraged lectin assistance to explore N‐glycosylation in both NSCLC sEVs and SCLC sEVs. A remarkable finding was the expression of integrin αV in both sEV types. However, a specific epithelium‐specific integrin, the α6β4 heterodimer, was uniquely identified in NSCLC sEVs, where it is modified by N‐glycans in NSCLC cells. This specificity paves the way for targeting N‐glycoproteins in cancer sEVs, offering new avenues for detection and potentially innovative therapeutic strategies.^243^

In the realm of medical research, not only is identifying biomarkers for diagnosis essential, but unraveling the intricacies of drug resistance plays a pivotal role in devising effective therapeutic strategies and evaluating patient outcomes. A key factor linked to drug resistance is abnormal alterations in protein glycosylation. A previous study revealed how increased N‐glycosylation of membrane proteins in NSCLC contributes to resistance to cisplatin, a commonly used chemotherapy drug.^244^ This study identified 297 membrane glycoproteins that exhibited differential expression between A549 cells and their cisplatin‐resistant counterparts, A549/DDP. Among these, 157 glycoproteins were found to be upregulated—primarily associated with cell adhesion and drug response—while 140 were downregulated, mostly related to cell signal transduction and migration.^244^ In addition, lectin nanoprobe‐based affinity mass spectrometry was applied to explore abnormal glycoproteins associated with lung cancer. This method was utilized to scrutinize glycoproteins enriched by MNPs coupled with aleuria aurantia lectin from the human lung adenocarcinoma cell line PC9. Through LC–MS/MS analysis, a notable discovery was made. Compared with the PC9 cell line, which is sensitive to gefitinib, the PC9 tyrosine kinase inhibitor‐resistant variant (PC9‐IR) exhibited a significantly higher level of fucosylation. This was particularly evident in the terminal fucosylation of EGFR at glycosite N413. These modifications hinder the normal interaction between the epidermal growth factor and its receptor, thereby affecting the ability of the receptor to form dimers. This, in turn, influences cell growth and the response to certain medications, including gefitinib.^245^

Abnormal glycosylation patterns are frequently observed during the progression of lung cancer, suggesting that the development of effective glycosylation inhibitors could be beneficial. A potential inhibitor, pictilisib, which utilizes network pharmacology and in silico screening techniques, was tested to assess the abundance of various N‐glycans, including high‐mannose, undecorated, fucosylated, sialylated, and sialo‐fucosylated glycans, in Pictilisib‐treated A549 cells. These results indicate that pictilisib treatment significantly reduced the overall relative abundance of fucosylated and sialylated N‐glycans. Notably, some of these glycans are involved in processes such as cell apoptosis, adhesion, and DNA damage repair. When treated with pictilisib, glycoproteins such as alanyl aminopeptidase, a disintegrin and metalloprotease 10, integrin beta‐1, and integrin alpha 3, exhibit reduced levels of fucosylation, sialylation, and sialofucosylation at specific glycosites. These glycoproteins are notably enriched in pathways related to cell adhesion, apoptosis, DNA damage responses, and cellular reactions to chemical stimuli.^246^

In conclusion, recent discoveries regarding the mechanisms of lung cancer, potential biomarkers for diagnosis and treatment at the glycosylation level, and advancements in glycoproteomics are both surprising and encouraging.^247^, ^248^, ^249^ However, various factors, such as inadequate sample sizes and inconsistent inspection standards, hinder the acceptance of glycoprotein detection as a widely recognized laboratory method for diagnosing lung cancer. Furthermore, accurately identifying the subtype of lung cancer is crucial for selecting appropriate treatments and predicting patient prognosis, and glycoproteomics can play a significant role in this process. Ideally, different components of glycoproteins would allow for precise verification of subtypes, but the current data remain insufficient for reliable diagnosis.^249^ Admittedly, there is still much work to be done in uncovering and illustrating the complexities of glycosylation, including the precise glycosylation and biological functions related to lung cancer. Advancements in this area hold the potential to provide significant benefits to lung cancer patients.

#### 3.3.2 Colorectal cancer

CRC represents a major health challenge globally and affects the colon and/or rectum. It ranks among the most prevalent cancers worldwide, leading to significant morbidity and mortality. Historically, CRC has predominantly affected middle‐aged and elderly individuals, particularly those aged 50 years and above. However, recent statistics indicate a worrying trend: an increase in the incidence of CRC among younger populations.^250^, ^251^, ^252^ In response, the medical community has expanded its arsenal beyond traditional treatments such as surgery, chemotherapy, and radiotherapy. Innovations in immunotherapy and molecular targeting therapies have been introduced, aiming to improve treatment outcomes. These advanced strategies focus on modifying the tumor microenvironment (TME) or directly inhibiting the proliferation of cancer cells, thereby offering a more efficient and effective approach to combating CRC.^253^, ^254^, ^255^

Through the application of advanced quantitative glycoproteomic techniques, a significant number of N‐glycoproteins have been identified in CRC tissue. This includes the discovery of nine glycoproteins that are markedly upregulated and linked with critical biological processes such as cell signal transduction, cell adhesion, and cell migration.^256^ In a separate investigation, an additional 160 glycoproteins whose expression was upregulated were identified. Among these, notable examples include intercellular adhesion molecule 1 (ICAM1) and adipocyte plasma membrane‐associated protein (APMAP).^257^ ICAM1 plays a pivotal role in various cellular functions, including proliferation, differentiation, death, apoptosis, and angiogenesis.^258^ Moreover, the presence of APMAP is correlated with hepatic metastases.^259^ These findings underscore the complex molecular mechanisms involved in CRC and highlight potential glycosylation targets for therapeutic intervention and research.

Exosomes contain a wealth of biological information that is ripe for investigation. One study utilized glycoproteomic techniques to compare the types and levels of glycoproteins in CRC tissues with those in normal tissues. The findings revealed that the levels of fibrinogen beta chain and beta‐2‐glycoprotein 1 in CRC‐derived exosomes were significantly greater than those in adjacent noncancerous tissues.^260^ In addition to N‐glycosylation, targeted O‐glycoproteomics has also proven valuable in identifying potential diagnostic markers for CRC. Takakura et al.^261^ reported that the O‐glycosylation patterns of glycoproteins are markedly elevated in the serum of CRC patients, suggesting new diagnostic approaches for this disease. In addition to glycoproteins, glycosyltransferases play crucial roles in CRC glycosylation, further emphasizing the complexity and potential of this research area in cancer diagnostics.^262^ Core 1 β1,3‐galactosyltransferase (C1GALT1), a critical enzyme in the mucin‐type O‐glycosylation process, functions as a type II transmembrane glycoprotein. This glycosyltransferase participates in the addition of a Gal to the Tn antigen, transforming it into the T antigen and continuously synthesizing complex O‐glycans, with the help of the chaperone protein Cosmc.^263^ Under normal physiological conditions, the Tn antigen is seldom observed due to its rapid conversion to the T antigen. However, in the context of cancer, particularly in tumor mucins, there is a notable increase in the presence of the Tn antigen. The overexpression of C1GALT1 not only alters the O‐glycan profile of the tyrosine kinase receptor, fibroblast growth factor receptor 2 (FGFR2) in CRC but also amplifies receptor activation by basic fibroblast growth factor.^264^, ^265^ This heightened activation contributes to cancer invasiveness and the potential for malignant metastasis. In contrast, the beta‐1,4‐N‐acetyl galactosaminyl transferase 2 (B4GALNT2) and its associated carbohydrate antigen, are abundantly expressed in normal colon tissue but are significantly reduced in CRC tissue. Intriguingly, patients with higher levels of B4GALNT2 mRNA have markedly better prognoses, highlighting the potential therapeutic importance of these glycosylation pathways in CRC management.^266^ In terms of the application in pathological level, glycoproteins serve a pivotal role in identifying the various subtypes of CRC. A notable study employed a porous graphitized carbon nano‐liquid chromatography system coupled to a mass spectrometer via electrospray ionization (PGC‐nano‐LC–ESI–MS/MS) to analyze the O‐glycosylation pattern among 26 different CRC cell lines, and unveiled the diverse range of O‐glycans present in these cells. Specifically, the research focused on the LS180 cell line, a human colon adenocarcinoma variant, revealing a high expression of sialyl Lewis x/a and Lewis x/a antigens. Conversely, the DLD‐1 cell line, characterized by its low differentiation in human colon adenocarcinoma, predominantly expressed core 2 sialylated glycans.^267^ In the study of CRC, particularly those identified as microsatellite instable (MSI), a notable alteration in their molecular composition has been observed. These tumors exhibit a decreased presence of α2‐3 sialylation and core 1 O‐glycans, while simultaneously showing an increased expression of the sialyl‐6T antigen. Furthermore, the mucinous adenocarcinomas in the T2 and T3 stages, have been found to express a unique set of 43 specific O‐glycans. These glycan structures predominantly carry the sLeX/A antigen, distinguishing these stages with a molecular signature. The evaluation of metastasis, particularly in the advanced Dukes C and D stages of invasive cancer, reveals an overexpression of sLeX/A antigens and α2‐3 sialylation on core 2 O‐glycans. This molecular feature offers promising avenues for the precise staging and treatment of tumors.^268^

In the discussion presented, glycoproteomics has significantly contributed to understanding CRC by identifying potential targets and elucidating the biological alterations associated with abnormal N/O‐glycosylation patterns. The identification of numerous abnormal glycosylations has been linked to the malignant progression of CRC. These findings not only broaden our understanding of CRC but also provide valuable diagnostic and therapeutic targets for patients.^269^ However, the inherent heterogeneity of tumors makes deciphering the complex glycosylation patterns associated with CRC challenging. Additionally, current clinical research is often limited by small sample sizes, making the findings less convincing and widely accepted. Therefore, future studies should utilize large, well‐characterized samples to thoroughly and systematically explore the glycoproteomic biological characteristics of CRC. This approach, combined with other advanced diagnostic methods such as gene detection, will pave the way for advancements in precision medicine.

#### 3.3.3 Liver cancer

Liver cancer ranks among the cancers with the highest mortality rates, predominantly because most patients are diagnosed at an advanced stage, which often leads to a less‐than‐ideal prognosis. The vast majority of liver cancers are classified as hepatocellular carcinomas (HCCs). The treatment for these carcinomas typically involves chemotherapy and/or immunotherapy.^270^ Recent epidemiological studies have identified obesity, hepatitis, alcohol consumption, smoking, and diabetes as the primary risk factors for LC.^271^, ^272^, ^273^ These factors contribute to the activation of inflammatory factors and the immune system in the liver, including the production of abnormal glycosylated proteins.^274^

In China, hepatitis B virus (HBV) infection stands out as one of the primary risk factors for HCC.^275^ A detailed analysis of HCC tissues and surrounding tissues revealed that, in comparison with adjacent nontumor tissues, there is notable dysregulation of fucosyltransferases in tumor tissues. This dysregulation is associated with an increase in fucosylated N‐glycans, which are prominently observed in glycoproteins such as IgA1 and IgG.^276^ Furthermore, similar findings have been reported for Hp in HBV‐related HCC, highlighting a consistent pattern across different studies.^277^ Expanding on this research, Li et al.^278^ have made significant strides by examining EVs in urine samples from HCC patients. Their work identified 344 distinct N‐glycopeptides that are expressed differently in HCC patients, with 36 being downregulated and a staggering 308 showing upregulation.^278^ Among them, the asparagine‐linked glycosylation protein 1 homolog is involved in the primary step of N‐glycosylation.^279^ Furthermore, investigations have explored the effects of epithelial–mesenchymal transition (EMT) inducers, such as TGF‐β, on fibrogenesis and carcinogenesis in hepatic cells, revealing an increase in these processes.^280^ Treatment with hepatocyte growth factor led to EMT‐like alterations in two types of HCC cells, SMMC‐7721 and HepG2. In these cells, nearly 80 glycans were identified at the N69 glycosite of folate receptor alpha (FOLR1), highlighting the intricate relationship between glycosylation processes and cancer progression. Further analysis revealed an increase in the core‐fucosylation of FOLR1 during the EMT process. An evaluation of the sole core‐fucosylation enzyme, fructosyltransferase (FUT8), revealed that the content of FUT8 was positively correlated with the core‐fucosylation level and folate uptake ability of FOLR1, which might provide novel biomarkers for HCC diagnosis and treatment.^281^

In the realm of HCC research, not only is the study of biomarkers critical but also the exploration of glyco‐biomarkers linked to the metastasis of HCC is highly important. A study utilizing advanced quantitative analysis technology for the examination of N‐glycoproteins in extrahepatic metastases, in contrast to nonmetastatic samples, revealed a notable alteration in N‐glycosite occupancy across 11 types of glycoproteins. These glycoproteins, including ceruloplasmin (CP), fibulin‐1, and galectin‐3‐binding protein, play crucial roles in cell adhesion and migration. They have been identified as potential biomarkers of cancer cell metastasis and indicators of poor prognosis.^282^, ^283^, ^284^ Furthermore, the p38 MAPK and NF‐κB pathways are consistently present in tumor tissues.^285^ Additionally, solute carrier family 35 member A2 (SLC35A2), which facilitates the transport of UDP‐galactose (Gal) from the cytosol to the Golgi apparatus or ER and is involved in glycosylation, is increased in HCC tissues. This increase is linked to the ability of cancer cells to invade, adhere, metastasize, and alter the membrane glycan profile, underscoring the complex interplay of glyco‐biomarkers in the progression of HCC.^286^, ^287^

In conclusion, the prevailing method for diagnosing and classifying liver cancer primarily relies on the measurement of AFP and a range of functional enzymes emanating from the liver.^288^ However, the accuracy of these biomarkers in diagnosis remains a matter of concern. Consequently, the identification of new biomarkers that could aid in clinical diagnosis is urgently needed. Furthermore, gaining deeper insight into the mechanisms underlying liver cancer will significantly enhance therapeutic approaches, particularly at the molecular level, which involves various glycoproteins. Currently, treatments often prioritize the use of sorafenib and/or mAbs, such as nivolumab and pembrolizumab, as frontline medications. Nonetheless, the efficacy of these treatments is somewhat limited, with some patients exhibiting noticeable resistance to these drugs.^270^, ^289^, ^290^ Therefore, it is imperative to investigate potential targets and develop innovative medications that can effectively curb the proliferation of HCC.

#### 3.3.4 Pancreatic cancer

PDAC ranks among the most prevalent pancreatic cancers globally, and is characterized by swift progression and a high fatality rate. Mechanically, metabolic reprogramming is the crucial hallmark of PDAC, along with the formation of a complicated and heterogeneous TME, laying the foundation for drug resistance.^291^, ^292^ The risk factors for PDAC can be include as family history, infection, diabetes, smoking, and obesity.^293^, ^294^ The diagnosis of PDAC typically involves a combination of clinical assessment, biopsy, imaging studies, and the evaluation of tumor biomarkers, including carbohydrate antigen 19‐9 (CA19‐9), carbohydrate antigen 242 (CA242), carbohydrate antigen 125 (CA125), carcinoembryonic antigen (CEA), and K‐RAS gene mutations.^295^ The primary approach to PDAC treatment integrates surgery with systemic chemotherapy.^296^, ^297^ Nonetheless, the pursuit of more accurate and effective diagnostic and therapeutic strategies has led to the exploration of immunotherapy and molecular targeting treatments, alongside the identification of various glycoproteins.^298^ CA19‐9, a useful biomarker, is often elevated in the serum of the majority of patients with pancreatic cancer. This biomarker plays a pivotal role in accelerating the progression of pancreatic cancer through various mechanisms such as glycosylation, interaction with E‐selectin, enhancement of angiogenesis, and involvement in the immune response.^299^ The formation of CA19‐9 (sialyl‐Lewis^a^) involves the gradual addition of sugar moieties to precursors for the α1,4 linkage of fucose to GlcNAc. This specific process is facilitated by a single enzyme, fructosyltransferase 3 (FUT3), which is responsible for the addition of moieties via an α1,4 linkage, thereby leading to the formation of CA19‐9.^300^ However, this enzyme is notably absent in mice, presenting a challenge for research in these models. To address this gap, an experiment to induce the expression of human FUT3 and β1,3‐galactosyltransferase 5 (β3GALT5) in mice, aiming to artificially synthesize CA19‐9 within their systems, was performed. Following induction, mice exhibiting elevated levels of CA19‐9 initially experienced acute and chronic pancreatitis, which was marked by an increase in inflammatory factors. Moreover, a positive correlation was observed between increase CA19‐9 levels and the over‐activation of pancreatic cancer progression mechanisms.^301^ Admittedly, the diagnostic process can sometimes be impeded by the occurrence of false positive or negative results when CA19‐9 levels are lilied upon. To increase diagnostic precision, it becomes imperative to identify additional biomarkers from patient samples. A study leveraging MALDI–MS technology investigated the IgG serum galactosylation profiles in patients with pancreatic carcinoma and those with benign pancreatic diseases, all of which had negative CA19‐9 results. The findings revealed that the level of IgG galactosylation in the pancreatic carcinoma group was significantly greater than that in the control group. Furthermore, the accuracy of diagnoses was markedly improved when CA19‐9 levels were evaluated in conjunction with IgG galactosylation levels.^302^ This approach underscores the potential of combining multiple biomarkers to refine the diagnostic process for pancreatic diseases.

Analyzing and comparing glycoproteome profiles from nonmetastatic and metastatic samples is crucial for selecting appropriate therapies and predicting patient prognosis. Using quantitative glycoproteomics, Wu et al.^303^ reported that 22 glycopeptides were upregulated in the plasma samples of pancreatic cancer patients, among which fucosylated serpin A1 (SERPINA1) was notable. Their research revealed a significant relationship between fucosylated SERPINA1 levels, the stage of pancreatic cancer, and patient prognosis. Specifically, elevated levels of fucosylated SERPINA1 correlated with increased tumor node metastasis stage and were associated with poorer prognosis and overall survival. This discovery highlights the potential of fucosylated SERPINA1 as a novel biomarker for diagnosing pancreatic cancer.^303^ Integrative proteomics and N‐glycoproteomics have emerged as valuable tools for assessing drug efficacy against pancreatic cancer.^304^ In one study, two pancreatic cancer cell lines, PANC‐1 and BxPC‐3, were treated with gemcitabine, aspirin, or a combination of both agents. Notably, the addition of aspirin inhibited the activity of these cell lines. To elucidate the underlying mechanism of this effect, a comprehensive quantification of intact N‐glycopeptides was performed. This analysis revealed a significant synergistic interaction between aspirin and N‐glycosylation. It was hypothesized that aspirin influences the sialylation and high‐mannose modification of lysosomal‐associated membrane protein 1/2 (LAMP1/2), thereby restraining cell autophagy and enhancing the efficacy of gemcitabine. In conclusion, N‐glycoproteomics offers a novel perspective for understanding the therapeutic targets associated with pancreatic cancer and the mechanisms of drug resistance.^304^

In summary, advancements of glycoproteomic technology have led to the identification of numerous glycoprotein targets, which are crucial for elucidating the mechanisms underlying pancreatic cancer and developing effective treatments. A notable correlation between abnormal glycosylation and pancreatic cancer has been established, with certain biomarkers recognized as indicators of disease progression.^305^ However, the heterogeneity of pancreatic cancer complicates the exploration of its glycoproteomic landscape. Additionally, the loss and degradation of glycoprotein structures during MS further contribute to the uncertainty of the results.^30^, ^306^ Therefore, it is imperative to pursue more in‐depth research to understand the causes and functions associated with this cancer, as such efforts will undoubtedly yield significant benefits for clinical treatment. The field of glycoproteomics has yielded significant discoveries, providing deeper insights into cancer biology. For example, current research focuses on assessing glycosylation levels in tumor cells, identifying aberrant glycosylation of proteins, and understanding the activation of related enzymes. Only a limited number of clinical diagnostic biomarkers, including AFP, PSA, ECA, and CA19‐9, have received approval from the US FDA.^307^, ^308^ Many glycoprotein markers remain under investigation. However, the accuracy of most existing markers requires further validation in future studies. Consequently, definitive diagnoses still depend on biopsy and pathological analysis, which are complex and not easily implemented on a large scale. Therefore, collecting samples such as serum, urine, and saliva for glycan profiling is a more convenient approach. With the integration of advanced algorithms, the widespread application of glycoproteomic diagnostic methods is anticipated to become a reality in the future.

### 3.4 Kidney diseases

#### 3.4.1 IgA nephropathy

**FIGURE 6:**
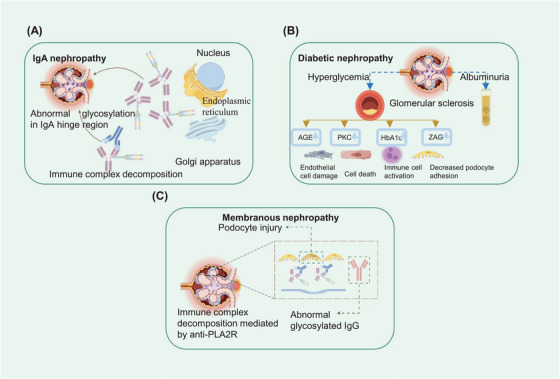
Simplified schematic of the possible pathogenesis of kidney diseases. (A) Pathogenesis of IgAN. (B) Pathogenesis of DN. (C) Pathogenesis of MN (by Figdraw). IgAN, IgA nephropathy; DN, diabetic nephropathy; AGE, advanced glycation end products; PKC, protein kinase C; HbA1c, hemoglobin; ZAG, zinc‐alpha‐2‐glycoprotein; MN, membranous nephropathy; PLA2R, phospholipase A2 receptor; IgG, immunoglobulin G.

The mechanism of IgAN remains unclear, yet the current understanding suggests that an elevated level of Gal‐deficient IgA1 (Gd‐IgA1) plays a significant role in its development.^309^ Additionally, activation of the complement system may contribute to the pathological accumulation of immune complexes.^310^ Figure 6A succinctly illustrates the connection between IgAN and glycosylation processes.

In a comprehensive review, analytical data from 22 studies were examined to assess the levels of Gd‐IgA1. The findings revealed that patients with IgAN presented significantly elevated concentrations of Gd‐IgA1 in their serum compared with healthy controls (*p *< 0.00001).^311^ IgA1 and IgA2, integral members of the IgA family, are glycoproteins. Notably, altered IgA1 was associated with reduced levels of Gal and/or sialic acid (NeuAc), and an increase in GalNAc.^312^ Furthermore, IgA2 has been shown to provoke greater release of neutrophil extracellular traps, thereby increasing the capacity to trigger inflammation.^313^ Glycoproteomic methods are also pivotal in exploring alterations in glycoproteins derived from human urine samples, offering insights into abnormal glycosylations. A notable study by Dotz et al.^27^ compared urine samples from 83 IgAN patients with those from 244 healthy controls. This comparison aimed to detect variations in N‐ or O‐glycosylated IgA1 and IgA2. LC–MS analysis revealed that changes in glomerular function are intricately linked to the diversity and complexity of N‐glycosylation processes. Specifically, abnormal O‐glycosylation of IgA1 has negative effects on glomerular function, and glycopeptide levels stand out as a precise indicator for predicting the onset of IgAN.^27^ The advancement of glycoproteomic technologies has also enabled researchers to identify glycoproteins that are either upregulated or downregulated, paving the way for the development of novel diagnostic tools for assessing renal tubular function.^50^ For example, researchers discovered 11 urinary glycoproteins uniquely present in IgAN patients. These glycoproteins could serve as potential diagnostic biomarkers for future medical practices.^314^ Similarly, an automated sandwich immunoassay system has been utilized to accurately measure the production of glycosylated IgA1.^315^ The use of wisteria floribunda agglutinin (WFA), which specifically binds to terminal GalNAc, allows for the identification of aberrantly glycosylated serum IgA1. This method, which employs WFA in a sandwich immunoassay format alongside a chemiluminescent enzyme immunoassay system, offers a reliable approach for glycan analysis.^315^, ^316^ In addition, Yu et al.^317^ reported that lower levels of GalNAc in the hinge region of IgA1 in IgAN patients are associated with different subtypes of the disease. An analysis of purified polymeric IgA1 via LC–MS/MS revealed that the levels of GalNAc3 and GalNAc4 were significantly higher in crescentic IgAN patients, while these levels were notably lower in healthy individuals. Conversely, GalNAc5 and GalNAc6 were found to be the lowest in patients with crescentic IgAN, slightly higher in those with noncrescentic IgAN, and the highest in healthy individuals.^317^ In 2001, Nakazawa et al.^26^ utilized MALDI–TOF–MS to examine IgA1 from 290 renal biopsy samples from 278 IgAN patients and from four serum IgA1 samples. Their findings revealed that the weight of circulating serum IgA1 was lower due to the lower presence of O‐glycan carbohydrates.^25^ Similarly, Nakazawa et al.^26^ reported that decreased O‐glycosylation (GalNAc and Gal) could be a critical contributor to the development of IgAN. Further research revealed variations in the expression of O‐glycosylation within the hinge region of IgA1 across three groups, healthy controls, non‐IgAN patients, and IgAN patients, and IgA affinity beads were used to assess the reliability of these biomarkers for diagnosis. The outcomes indicated that GalNAc levels were significantly lower in patients with IgAN, suggesting its potential as an effective biomarker for this condition.^318^

As discussed above, IgA1 with abnormal O‐glycosylation is recognized by IgG and binds in a distinctive manner. This interaction suggests that separating IgG from patients with IgAN and introducing Gal‐IgA1 could prompt the formation of an immune complex. In support of this hypothesis, the use of specific IgG and Gal‐IgA1 in a glycan‐dependent approach successfully generated an immune complex, which is instrumental in assessing the progression of the disease.^319^ Moreover, glycosyltransferases play pivotal roles in the O‐glycosylation process. A particular study utilized LC–MS to explore the correlation between glycosyltransferase enzymatic activities and the biosynthesis of O‐glycans on IgA1.^320^ This study primarily assessed the process of the polypeptide N‐acetylgalactosaminyltransfersase reaction and the resulting products. This investigation simulated the O‐glycosylation procedure through successive carbohydrate additions, highlighting the crucial mediating role of specific glycosyltransferases.^320^

Overall, recent findings have focused predominantly on the reduction in O‐glycosylated proteins as key biomarkers for revealing the progression of IgAN. Statistical analysis of clinical samples has revealed a correlation, yet the mechanism by which these uniquely expressed glycoproteins, alongside other potentially abnormally expressed glycoproteins, such as those in the complement system, contribute to the onset of IgAN remains unclear.

#### 3.4.2 Diabetic nephropathy

Diabetic nephropathy (DN), a secondary injury caused by prolonged hyperglycemia, represents a significant challenge in the medical community. This condition, characterized by secondary injuries to the kidney, is driven primarily by microinflammation and the expansion of extracellular matrices, both of which are exacerbated by elevated blood sugar levels.^321^ Modern researchers have encountered significant hurdles in both the accurate detection of conditions such as nonalbuminuria and the development of precisely targeted treatments.^322^ As a result, the scientific focus has shifted toward the study of diabetic glycoproteins, offering a promising avenue for both diagnosis and intervention.^323^ This exploration holds the potential to revolutionize our approach to managing diabetic kidney injuries, marking a critical step forward in the battle against DN.

Two‐dimension differential LC–MS/MS (2D‐LC–MS/MS) alongside isobaric tags for relative and absolute quantitation was used to examine glycoprotein variations across different stages of type 2 DN, including normal controls, normoalbuminuria, microalbuminuria, and macroalbuminuria conditions.^324^ This comprehensive study identified 408 distinct glycoproteins. Specifically, the analysis revealed 72, 107, and 123 glycoproteins uniquely present in the normoalbuminuira, microalbuminuria, and macroalbuminuria groups, respectively. The findings of the present study demonstrated that both cell death and apoptosis were significantly triggered in the microalbuminuria and macroalbuminuria groups. Notably, within the macroalbuminuria group, there was a marked increase in the levels of acute‐phase proteins such as serpin family A member 1 (SERPINA1), CP, and transthyretin. This elevation suggests that renal inflammation and fibrosis occur within the kidneys. Such a trend points toward the potential of these glycoproteins as novel biomarkers for the diagnosis of kidney‐related conditions.^324^ Another study aimed to discern the differences in the kidney N‐glycoproteome between normal and diabetic mice.^325^ To achieve this goal, models of diabetes were established via the use of db/db mice treated with insulin to induce type 2 diabetes and mice treated with streptozotocin (STZ) to induce type 1 diabetes. The investigation of the N‐glycosylation profiles revealed notable differences between the two types of diabetes. Although certain glycoproteins, such as HbA1c, exhibited similar increases in both models, integrin‐β1 showed a divergent pattern, increasing in STZ‐induced diabetic mice but decreasing in db/db mice. Conversely, SGLT1 has been shown to have the opposite effect.^325^ Furthermore, zinc‐alpha‐2‐glycoprotein (ZAG) has been identified as a critical biomarker for the early detection of kidney injury in individuals with type 2 diabetes mellitus. Its significant elevation in both urine and serum suggests the potential of ZAG to predict early DN, particularly in stages without albuminuria. This is underscored by its inverse relationship with the estimated glomerular filtration rate.^326^, ^327^ Therefore, ZAG could be a novel biomarker for nonalbuminuric DN.^328^, ^329^ Additionally, a study employing SNA blot analysis revealed that Sia α2‐6 Gal/GalNA is markedly present in the urine of patients with DN. Moreover, the levels of this glycopattern are directly correlated with the progression of DN.^330^ The integrity of the glomerular endothelial cell surface is crucial for preventing albumin leakage. Research has shown that proteoglycans, which are proteins that attach one or more glycosaminoglycan chains to a core protein, are vital for maintaining the integrity of the glomerular endothelial cell surface.^331^ This is achieved by the formation of a layer on the luminal cell surface. Figure 6B succinctly summarizes the findings related to glycosylation in DN.

Current research offers preliminary insights into identifying predictors for early DN. However, the sample sizes used in these studies are still not sufficient to draw definitive conclusions. Additionally, the specific biological functions of these glycoproteins remain unclear, highlighting the need for further research to elucidate their roles.

#### 3.4.3 Membranous nephropathy

The majority believe that primary membranous nephropathy (PMN) or idiopathic membranous nephropathy (IMN) is a kidney‐specific, autoimmune glomerular disease characterized by increased urine protein and continuous glomerular injury. It is often caused by antibodies against the M‐type phospholipase A2 receptor (anti‐PLA2R), leading to the accumulation of autoimmune complexes in the glomeruli, including complement.^332^, ^333^ Furthermore, under immunofluorescence staining, one can observe small granular deposits of IgG (particularly IgG4 in the PMN) in subepithelial locations, accompanied by the loss of podocyte foot processes.^334^, ^335^ Figure 6C summarizes the possible pathogenesis associated with glycosylation in MN.

Research has revealed that the altered glycosylation of IgG4 plays a role in advancing the progression of PMN.^336^ Serum containing IgG4 from PMN patients has been shown to trigger the proteolysis of the podocyte proteins synaptopodin and nephrin‐related protein 1, disrupting the structure of the podocyte cytoskeleton. Moreover, the anti‐PLA2R1 IgG4, in a glycosylation‐dependent manner, is capable of binding with mannose‐binding lectin (MBL), thereby activating the lectin complement pathway and subsequently damaging podocytes. This insight opens a novel avenue for PMN treatment, specifically targeting and blocking the lectin pathway.^336^ Moreover, building on the strong correlation between MN and PLA2R, a study utilized an N‐glycan purification technique known as ultrafast glycoprotein immobilization for glycan extraction.^337^, ^338^ This method revealed that in the PLA2R1‐positive IMN group, sialylation and core‐fucosylation were upregulated, whereas galactosylation was downregulated. On the basis of these findings, the analysis introduced a six‐glycan marker panel (H4N3S1, H4N3F1, H6N4S2, H6H5F1S2, H6N5, H6N6F1S1), which contributes to diagnosis of patients with PAL2R1‐related IMN.^337^, ^338^ Figure 6C provides a concise overview of the discoveries of glycosylation in MN. Compared with those related to IgAN and DN, there are significantly fewer discoveries related to MN. One contributing factor is the lack of sufficient research into the mechanisms of this disease. Without a needle biopsy, achieving an accurate diagnosis and treatment poses a challenge for clinicians.^339^, ^340^ Similarly, pathologists also face difficulties in making diagnoses at the pathological level. Therefore, it is imperative that more effort is directed toward this field and that additional biomarkers be identified to ease the challenges of diagnosis.

In conclusion, the field of clinical glycoproteomics has achieved significant progress in understanding CKDs. Despite these advancements, the path forward is filled with challenges and unanswered questions. Most research on glycoproteomics has concentrated on comparing samples from CKDs. This strategy has been crucial in discovering potential biomarkers for different kidney diseases. However, the clinical relevance of these biomarkers is still somewhat unclear. A major problem is that variables within the study populations can distort the results, affecting the reliability of the findings. Furthermore, the causal relationship between glycoproteins and CKDs remains largely unexplored. This gap in knowledge casts a shadow of doubt over the clinical implications of glycoproteins that are either overexpressed or underexpressed in patients. Additionally, while glycoproteomic data can emerge from genome‐wide studies, the challenge of integrating these data persists owing to a lack of comprehensive datasets. This limitation impedes the ability to conduct thorough analyses that could reveal deeper insights into the disease mechanisms at play. Another critical aspect to consider is the spatial heterogeneity introduced by analyzing glycoproteins through blood and urine tests. This method offers a glimpse into changes occurring within specific organs but does not provide a full picture of the systemic alterations in the body.

Viewing diseases in isolation can hinder our understanding of their progression and overall impact on the body. There is a growing belief in the importance of integrating additional clinical biomarkers, such as kidney injury molecule‐1 (KIM‐1), β‐Trace protein, neutrophil gelatinase‐associated lipocalin, and N‐acetyl‐β‐d‐glucosaminidase, to increase diagnostic accuracy.^341^ Furthermore, some experts are combining genomics, proteomics, transcriptomics, metabolomics, and glycoproteomics, which are rooted in molecular mechanisms, to aid in the clinical diagnosis of CKDs and establish specific classifications and treatments.^342^, ^343^ Although we identified numerous molecules that facilitate the identification of CKD subtypes with less invasive examinations, a standardized criterion for accurate subtype diagnosis is still lacking. Therefore, it is crucial to discover new biomarkers and elucidate their interactions, establishing their connections with these mechanisms. Notably, all these techniques aim for precise diagnosis and improved targeted therapeutic strategies. In summary, in the field of clinical glycoproteomics, addressing the current challenges and bridging the existing knowledge gaps are essential.

### 3.5 Metabolic diseases

#### 3.5.1 Diabetes

**FIGURE 7:**
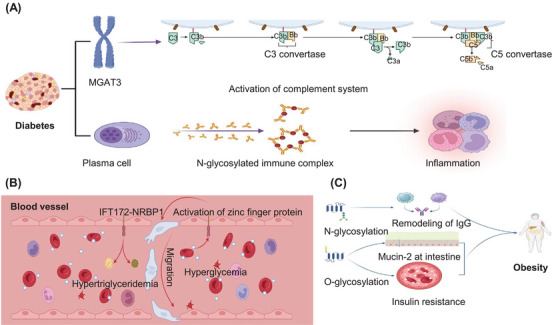
Pathological role of glycoproteins in diabetes and obesity. (A) N‐glycoproteins are associated with the activation of the complement system and inflammation triggered by immune complexes. (B) Influence of O‐glycosylation on circulation during hypertriglyceridemia and hyperglycemia. The zinc finger glycoprotein in endothelial cells is activated by N‐acetylgalactosaminyl transferase 14, which further enhances the migration of endothelial cells. The O‐glycosylation of IFT172/NRBP1 region is associated with hypertriglyceridemia. (C) Two distinct pathways of O‐glycosylation and N‐glycosylation in obesity. IgG undergoes remodeling through the addition of N‐glycans, while O‐glycans affect intestinal permeability and insulin resistance (by Biorender). MGAT3, beta‐1,4‐mannosylglycoprotein 4‐beta‐N‐acetylglucosaminyltransferase; IFT172, intraflagellar transport 172; NRBP1, nuclear receptor‐binding protein 1.

In recent years, the field of diabetic glycobiology has played a significant role in both the diagnosis and treatment of diabetes. Glycated albumin, HbA1c, and other glycated proteins have been shown to influence cellular functions, transport mechanisms, and immune responses, all under the clinical supervision of diabetes management.^344^, ^345^, ^346^ However, detailed information regarding the structures and functions of these glycoproteins in the pathogenesis of diabetes remains unclear. With the advancement of glycoproteomic techniques, the glycosylation landscape, particularly the typical N‐glycosylation and O‐glycosylation, has begun to be elucidated, providing a deeper understanding of the complexities associated with diabetes (Figure 7A).

The analysis of glycopeptides using LC–MS among 61 children with type 1 diabetes and 84 healthy siblings confirmed a positive association between type 1 diabetes and alterations in plasma high‐mannose glycans.^347^ Specifically, this investigation revealed a significant overall decrease in monogalactosylated glycans, accompanied by increases in digalactosylated, monosialylated, and antennary fucosylated‐derived N‐glycosylation.^348^ Additionally, GWASs have pinpointed the glycosyltransferase gene that encodes fucosyltransferase 2 as a susceptibility gene linked to type 1 diabetes.^349^ A comprehensive study integrating glycomics and genetics, involving 1105 recent‐onset type 1 diabetes patients in Denmark, identified the candidate gene MGAT3 and an allele of two C3 SNPs as novel genetic associations influencing N‐glycated Igs in type 1 diabetes (Figure 7A).^350^ Integrative glycoproteomic analyses indicated that hyperglycemia promotes the N‐glycosylation of Igs. The onset of type 1 diabetes has been closely linked to an increase in the proportion of plasma IgG structures characterized by high‐mannose and bisecting GlcNAc forms, alongside a decrease in monogalactosylation and an increase in disialylation.^351^, ^352^ These changes suggest potential roles in anti‐inflammatory responses, the typical pathways of complement activation, and hormone release across different sexes. Based on these findings, the increase in autoimmune antibodies, coupled with a decrease in highly branched N‐glycans, demonstrated excellent predictive capability for type 1 diabetes, achieving an area under the curve of 0.915 after incorporating IgG N‐glycans.^351^

In the context of type 2 diabetes, N‐glycans has also been identified in plasma Igs. A study involving three prospective cohorts demonstrated a negative correlation between plasma IgG galactosylation and both DN and the incidence of retinopathy.^353^ Cardiovascular events represent a significant independent risk factor contributing to all‐cause mortality in individuals with type 2 diabetes. Recent researches have highlighted an association between cardiovascular events and N‐glycosylation in diabetes. For instance, two nested case‐control studies indicated that an agalactosylated glycan was positively associated with cardiovascular outcomes, whereas three digalactosylated glycans and two monosialylated glycans were negatively associated with cardiovascular events.^354^ Meanwhile, other researches have demonstrated a significant association between various IgG glycopeptides and the development of type 2 diabetes.^355^, ^356^ In addition to the aforementioned points, the complement system plays a crucial role in the onset and progression of type 2 diabetes. Research indicates that levels of N‐glycopeptides derived from complement C1s, mannose‐associated serine protease 1, and complement factor P decrease in high‐glucose environments, while those from complement C2, C4, C4BPA, C4BPB, and CFH proteins increase.^357^ This dysregulation of N‐glycopeptides in the activation of the complement system may hinder the formation of complement C3Bb C3 convertase and contribute to the loss of Ig biomarkers associated with type 2 diabetes (Figure 7A).

Alterations in N‐glycans have been proposed as a therapeutic strategy; however, such changes have not been consistently observed at the proteomic level. For example, a study demonstrated that metformin, the first‐line treatment for type 2 diabetes, effectively reduced fucosylation while increasing galactosylation and sialylation, independent of IgG levels.^358^ The shifts in N‐glycosylation as biomarkers for precise diagnosis should take medication into account as a potential confounding factor. In summary, N‐glycosylation primarily influences IgG and complement‐associated proteins, regardless of whether it is type 1 or type 2 diabetes. However, the current research predominantly presents a changing spectrum of N‐glycans in diabetes, while the biological role of dysregulated N‐glycosylation remains unclear. This aspect warrants further investigation in future studies.

In comparison, our understanding of the molecular structure and biological significance of O‐glycosylation in diabetes remains limited. Polypeptide N‐acetylgalactosaminyl transferase 14 (GALNT14), a crucial enzyme implicated in cancer progression, has been shown to regulate the growth of β cells and influence the dysregulation of O‐glycosylation in the progression of diabetes.^359^ In STZ‐induced diabetic mouse models, the expression of zinc finger ribonucleic acid binding protein was found to be abnormally elevated, which in turn facilitated the migration of human retinal microvascular endothelial cells.^360^ Moreover, apolipoproteins modified by O‐glycans have been linked to the promotion of hypertriglyceridemia in a high blood sugar environment. Genetic analyses indicated that glycosylated apo‐CIII, which can have zero, one, or two sialic acids (apo‐CIII0c, apo‐CIII1, and apo‐CIII2) at the IFT172/NRBP1 region, is positively associated with hypertriglyceridemia in diabetic patients.^361^ In the following year, mono‐sialylated apo‐CIII1, disialylated apo‐CIII2, and apo‐CIII0a were found to be correlated with diabetic retinopathy and macrovascular events (Figure 7B).^362^ Additionally, hypoglycemia induced a significant increase in endothelial nitric oxide synthase O‐GlcNAcylation, accompanied by a decrease in Ser‐117 phosphorylation in the thoracic aorta and mesenteric arteries.^363^ These alterations were more pronounced under insulin‐induced hypoglycemia, further exacerbating the impairment of endothelial‐dependent vasodilation. Furthermore, diabetic retinopathy is a common complication of diabetes, resulting from microvascular damage. Preclinical analyses have revealed elevated O‐glycosylation of syntaxin‐1A, syntaxin‐3, and complexin‐4 proteins involved in synaptic vesicle docking and fusion in the retinas of STZ‐induced diabetic mice. These evidences, although significant, remain incomplete, highlighting the need for further investigation into glycosylation in various diabetes‐related complications and its potential implications for diagnosis and treatment.

#### 3.5.2 Obesity

With the rise of sedentary lifestyles, the prevalence of obesity has dramatically increased worldwide, leading to a growing double burden in many countries.^364^, ^365^ Glycobiology has been shown to play a significant role in obesity. A study conducted in Australia utilized dual‐energy X‐ray absorptiometry to investigate a sample of 637 community dwellers, identifying 22 N‐glycan peaks in IgG that were associated with central adiposity, which in turn was linked to a more severe inflammatory response.^366^, ^367^ Due to the pronounced changes observed in individuals diagnosed with central adiposity, a dysregulated immune system may contribute to additional disorders in obese individuals. Furthermore, bacterial colonization factor‐1, which possesses N‐glycans, could promote the colonization of *E. coli*, potentially playing a role in maintaining intestinal homeostasis.^368^ Low‐calorie diet, a conventional approach to weight management, can lead to a reduction in high‐branched trigalactosylated and trisialylated plasma N‐glycans, along with a corresponding increase in low‐branched N‐glycans. In contrast, bariatric surgery results in a significant increase in high‐branched and antennary N‐glycans, while simultaneously causing a substantial decrease in simpler and low‐branched N‐glycans among patients with obesity.^369^ Furthermore, the chromatographic profiling of IgG indicates a marked decrease in IgG N‐glycans and bisecting GlcNAc following the loss of excess body fat, which contributes to a partial reduction in biological age and inflammation levels (Figure 7C).^370^ Furthermore, physical exercise is a vital strategy for managing obesity and has been shown to reduce body fat through the alteration of N‐glycans. When comparing professional competitive athletes to individuals who engage in moderate physical activity, those who are newly involved in recreational activities, and inactive individuals, the GlycanAge index, which reflects biological age, decreases with increased intensity of physical activity.^371^ A linear mixed model focusing on overweight individuals who engage in regular exercise revealed significant glycosylation remodeling, characterized by an increase in agalactosylated, monogalactosylated, asialylated, and core‐fucosylated N‐glycans, while there was a decrease in digalactosylated and mono‐ and di‐sialylated N‐glycans.^372^ Overall, these findings suggest that various obesity treatment methods (such as physical exercise, low‐calorie diets, and surgical interventions) can enhance metabolic function, potentially through the reprogramming of N‐glycosylation.

O‐glycosylation regulates obesity mainly through the maintenance of intestinal mucosal integrity and the modulation of insulin resistance. In studies involving B3galt5 knockout mice, the activation of the pregnane X receptor has been shown to enhance the expression of β−1,3 galactosyltransferase 5, an enzyme essential for sustaining the O‐glycosylation of mucin‐2 and the mucus layer (Figure 7C).^373^ This mechanism further exacerbates obesity induced by a high‐fat diet. Traditional herbal remedy Si‐wu has been found to improve O‐glycosylation and correct the protein folding of mucin‐2, thereby alleviating gut barrier damage in a high‐fat microenvironment.^374^ Regarding insulin resistance, cyanidin‐3‐O‐β‐glucoside has been reported to inhibit the O‐glycosylation of the transcription factor FoxO1, which subsequently reduces the expression of adipose triglyceride lipase (Figure 7C).^375^ Additionally, LC–MS/MS analysis of nitrosated proteins in erythrocytes has revealed a decrease in O‐glycosylation levels.^376^ It can be inferred that the decline in O‐glycan contributes to impaired erythroid antioxidant defenses via catalase activity in children suffering from obesity and insulin resistance. All these results indicate that N/O‐glycosylation is widely involved in the occurrence and development of obesity.

### 3.6 Other diseases

#### 3.6.1 Coronavirus disease 2019

**FIGURE 8:**
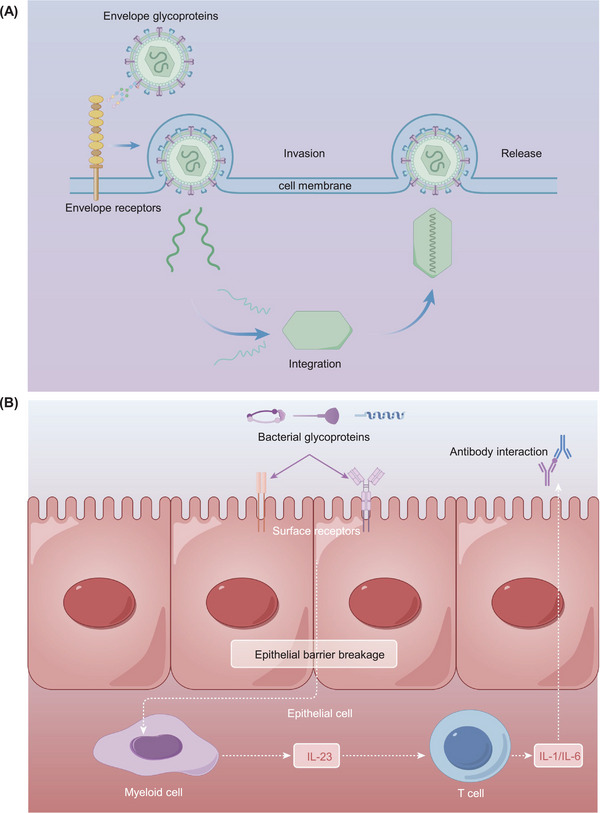
Glycoprotein interactions between pathogens and human tissues or cells. (A) Mechanism of coronavirus infection via glycoprotein binding. When the envelope glycoproteins of coronaviruses attach to receptors on the host cell membrane, the viral nucleic acid enters the cytoplasm, initiating intracellular replication by harnessing the resources of the host cells. (B) Mechanism of bacterial invasion through glycoprotein recognition. In systemic infections such as sepsis, bacterial glycoproteins disrupt epithelial barriers and are recognized by innate immune myeloid cells and T cells. This recognition subsequently triggers antibody responses mediated by cytokines (by Figdraw). IL, interleukin.

Since 2020, the global outbreak of coronavirus disease 2019 (COVID‐19), caused by severe acute respiratory syndrome coronavirus 2 (SARS‐CoV‐2) has tragically claimed millions of lives across the globe.^377^ The coronavirus enters host cells by attaching envelope (Evn) proteins (such as spike glycoproteins) to the corresponding receptors.^378^ The spike glycoprotein has carbohydrate components that cover the receptor‐binding domain (RBD), hiding it temporarily through hinge conformational movements. This clever mechanism allows the virus to evade the immune system and resist drugs (Figure 8A).^379^ The primary receptor targeted by the spike glycoprotein is the membrane angiotensin‐converting enzyme 2 (ACE2) on cell membranes. Once engaged, this interaction can lead to the fusion of the host cell with the virus.^380^ Notably, the interaction between the spike glycoprotein and ACE2 plays a crucial role in determining an individual's susceptibility to infection.^381^ Recent studies have identified several co‐factors that can act alongside ACE2, offering new insights into how the virus operates. As a result, carbohydrate components, including N‐glycans that comprise oligomannose and complex glycans that inhibit antibodies, have the potential to engage with lectin receptors.^382^, ^383^

Given the significant role glycoproteins play in disease mechanisms, glycoproteomic techniques have the potential to aid in diagnosis and monitoring. In 2022, long pentraxin 3 and MBL were shown to bind to the viral nucleocapsid and spike glycoprotein, respectively.^384^ MBL activates the lectin pathway of the complement system, assisting in the assessment of disease severity. Stewart et al.^385^ demonstrated that reducing the levels of intracellular tetherin led to an increase in SARS‐CoV‐2 viral loads. N‐glycosylation plays crucial roles in both the invasion of viruses and the regulation of immune responses. All 22 N‐glycosites on the spike protein were identified, which facilitated hemagglutination.^51^, ^386^ Amraei et al.^387^ discovered that the RBD of the SARS‐CoV‐2 spike glycoprotein interacts with the C‐type lectin CD209/DC‐SIGN and CD209L/L‐SIGN proteins. After removing the N‐glycans on the N92 glycosite, the combination of the spike RBD and CD209L/L‐SIGN intensified. These findings may provide a new strategy for antiviral drug development. Similarly, a panel of N‐to‐Q mutations at spike N‐glycosites (N61, N603, N657, and N616) was created.^388^ In particular, mutations in N61Q and N801Q significantly decreased pseudovirus entry by 80 and 90%, respectively. This finding was determined via flow cytometry, which revealed that inhibiting the dolichyl‐diphosphooligosaccharide‐protein glycosyltransferase subunit STT3A can reduce the external infectivity of the SARS‐CoV‐2 variant by encouraging N‐glycosylation deformation.^389^ Besides, three ACE2 glycosites (N53, N90, and N332) may serve as specific targets for interactions with the spike glycoprotein.^390^ Throughout the global spread of COVID‐19, researchers have observed changes in nine N‐glycoites of the spike glycoprotein without any mutation, suggesting new avenues for vaccine development.^391^, ^392^ Regarding humoral immunity, a genetic study pinpointed that N‐glycosylation on IgG could increase susceptibility to COVID‐19.^393^ In the analysis of antibody domains, N‐glycosylation is frequently found in antiviral antibodies. The substitution of polar residues (serine and threonine) to introduce N‐glycosites shows promise in modifying the biological functions of antibodies.^394^

In addition, IgM, an initiator of the response to viral infection, has a strong effect on downstream immune function. In 2024, an analysis of IgM N‐glycosylation revealed high levels of mannosylation and sialylation, which are significantly associated with disease severity.^395^ Furthermore, the IgM‐dependent accumulation of complement proteins was found to be elevated and was monitored through exoglycosidase digestion.^395^ In terms of O‐glycosylation, dozens of O‐glycosites have been identified, and 3 O‐glycosites were considered as vaccine targets.^55^, ^391^ Most O‐glycosites are modified by core‐1 and core‐2 O‐glycans.^396^ Zhang et al.^397^ discovered proline (P681) dependent O‐glycosylation, which may influence viral infectivity by modulating furin cleavage of the spike protein. Additionally,, sialic acid‐containing O‐glycans on threonine 678 of the SARS‐CoV‐2 spike protein negatively influence cleavage by furin and transmembrane protease serine 2, facilitating the evolution of variants of concern.^398^

On the basis of accumulating evidence, the investigation of viral particles and antigens presents a feasible alternative to traditional polymerase chain reaction (PCR) methods. These techniques can detect structural proteins of SARS‐CoV‐2, including the spike protein, membrane protein, and Env protein.^399^ Using swab samples from COVID‐19 patients, LC–MS/MS has been identified as a viable option for detecting SARS‐CoV‐2 glycoproteins.^400^ Cazares et al.^401^ reported a targeted MS technique that enables specific recognition of the SARS‐CoV‐2 spike protein and nucleoprotein within biological matrices, achieving a limit of detection of 200 amol, which indicates that identification can be accomplished at the single amino acid level. Similarly, Bezstarosti et al.^402^ achieved remarkable results, with a detection limit of 0.9 pg of nucleocapsid within just 1.5 h. The sensitivity of these methods is comparable to, or even exceeds, that of PCR, indicating promising potential for early diagnosis. Additionally, several studies have revealed that glycoproteomic analysis can systematically elucidate the severity of COVID‐19. For instance, fucosylated N‐glycans have been shown to be negatively correlated with the severity of COVID‐19.^403^ Concurrently, elevated levels of oligomannose‐ and sialylated di‐antennary glycans, along with decreased levels of nonsialylated di‐antennary glycan, may serve as biomarkers for the pathogenesis of COVID‐19.^404^

#### 3.6.2 Acquired immune deficiency syndrome

Human immunodeficiency virus (HIV) infection is generally characterized by the progressive depletion of cluster of differentiation 4 receptor (CD4+) T cells, ultimately leading to immune deficiency.^405^ Recent research indicates that the subtypes of the HIV are continuously evolving, presenting significant challenges for vaccine development and contributing to widespread transmission.^406^ Additionally, migration and globalization have further facilitated the global pandemic of this disease.^406^, ^407^ Like in COVID‐19, glycosylated spike proteins on the viral membrane significantly increase the ability of HIV to invade host cells.^408^ Over the past 30 years, recombinant HIV‐1 Env glycoproteins expressed on membranes have been considered promising candidates for vaccine development because their multiple epitopes can elicit neutralizing antibodies.^409^ However, continuous mutations in the virus lead to the emergence of various glycans, such as anti‐V1/V3‐glycans, which facilitate the identification of novel vaccine targets.^410^, ^411^ This evolving landscape underscores the importance of clinical glycoproteomic techniques in advancing vaccine research.

In 2018, N‐glycosylation at the glycosite N294 of an inhibitor of the retroviral infectivity inhibitor serine Incorporator 5 (SERINC5) was shown to increase the functionality of this protein, effectively preventing the reverse transcription of HIV.^412^ Keating et al.^413^ discovered a kind of self‐glycan addition under experimental conditions. After the pruning of N‐glycans by amidase, the major viral spike proteins automatically attach to the de‐N‐glycosylated sites. Previously, it was believed that HIV‐1 lacked O‐linked glycans on its Env; however, this conventional understanding was challenged in 2020. Research has revealed the O‐glycosylation in a subset of patient‐derived HIV‐1, which was found to be present both on virions and on gp120.^414^ Furthermore, these O‐linked glycans were shown to reduce the sensitivity of neutralized antibodies to one out of 1000, indicating a functional role in viral escape.

Clinical glycoproteomic techniques play crucial roles in the development of therapeutic strategies and diagnostic methods for HIV infection. It is well‐established that neutralizing antibodies have the potential to prevent HIV invasion. In 2020, Chuang et al.^415^ introduced a method for removing N‐linked glycosylation sequons from variable domains, which can significantly reduce chemical heterogeneity, thereby providing clearer and more concise targets for vaccine development. In addition, interferon‐alpha, a well‐known antiviral molecule, has been shown to bisect GlcNAc glycans, thereby promoting inflammation and altering the function of cluster of differentiation 8 receptor (CD8+) T cells in the context of the HIV immune response.^416^ The glycosylation of antibodies is indicative of their clinical function and application value. In three distinct cohorts, HIV‐specific antibody Fc N‐glycosylation was significantly associated with viral rebound following treatment discontinuation.^417^ Chen et al. developed a self‐assembled peptide nanofiber by modulating Fc N‐glycosylation.^418^ The vaccine combined with this nanofiber demonstrated enhanced tier 1 neutralization compared with the HIV Env antigen alone. On the basis of these findings, future vaccine development should incorporate glycoproteomic techniques to leverage glycol‐immunology targets in HIV infections.^419^

Immune escape mechanisms mediated by glycosylation pose significant challenges in the diagnosis of HIV infection. Various detection kits have been developed to identify antibodies against different components of HIV; however, accurately identifying HIV‐positive individuals at an early stage remains difficult.^420^ As previously mentioned, the glycosylations present on viral particles play crucial roles in immune evasion, resulting in a dynamic pattern of mutual growth and decline. Abbasi et al.^421^ employed a hydrophobic reaction to link gp120 with the RNA aptamer B40t77, resulting in a signal amplification of gp120 through liquid crystal (LC) technology, achieving a detection limit of 1 µg/mL after a 30‐min incubation period. This research continued in 2021, where a label‐free biosensing platform facilitated homeotropic alignment between B40t77 and gp120A.^422^ This platform was enhanced by LC methodologies, and the detection limit was reduced to 0.2 µg/mL. These findings leverage glycoproteomic techniques to foster innovative early diagnostic approaches for HIV. However, these results, while promising, are not yet widely implemented and require further validation.

#### 3.6.3 Sepsis

Sepsis is a life‐threatening condition characterized by multiple organ dysfunction resulting from host immune disorders.^423^ A nationwide study in Sweden estimated the morbidity of sepsis to be 747 cases per 100,000 individuals, with a median age of 76.^424^ The antigen‐antibody reaction accounts for a large part of the immune response associated with sepsis. In systemic infections, bacterial glycoproteins disrupt epithelial barriers and are recognized by innate immune myeloid cells and T cells. This recognition subsequently triggers antibody responses mediated by cytokines (Figure 8B). Investigations into the glycosylation of antibodies provide insights into their functions. Yaykasli et al.^425^ conducted an analysis of serum cytokine, Ig and neutrophil elastase concentrations. These results indicate that as sepsis progresses, there is an increase in neutrophil‐associated cytokines and elastase, driven by elevated fucosylation and α1,3‐galactosylation in the Fc region of IgG, as observed through LC–MS/MS. In the context of the mucosal barrier, sepsis induced by severe burns promotes the activation of S‐glutathionylation, further intensifying the modification of O‐linked GlcNAc (O‐GlcNAc).^426^ This process enhances the synthesis of nicotinamide adenine dinucleotidephosphate, thereby bolstering the antioxidative capacity during septic infections. Consequently, MS recognition facilitates the identification of adaptive diagnostic and prognostic biomarkers.^427^, ^428^ García‐Giménez et al.^429^ employed a mass spectrometry technique known as multiple reaction monitoring to analyze the levels of the circulating histones H2B and H3 in plasma. Researchers have reported that H2B levels exceeding 121.40 ng/mL are indicative of septic shock, whereas histones H2B levels exceeding 435.61 ng/mL and H3 levels above 300.61 ng/mL indicate the need for invasive organ support. Furthermore, glycoproteomic molecular approaches, such as PCR/electrospray ionization‐mass spectrometry (PCR/ESI‐MS), distinguish between gram‐positive bacteria, gram‐negative bacteria, and candida species in septic blood samples. This advancement significantly enhanced the molecular diagnosis and pathogenic classification of sepsis.^430^ Yu et al.^431^ combined the Rapid Sepsityper Kit with machine learning‐based MALDI–TOF–MS for the rapid prediction of methicillin‐resistant *Staphylococcus aureus* and carbapenem‐resistant *Klebsiella pneumoniae*. In the study involving 461 septic blood samples, 44 isolates of *S. aureus* and 126 isolates of *K. pneumoniae* were evaluated, yielding receiver operating characteristic values of 0.898 and 0.828, respectively. These findings offer a novel perspective on rapid detection methods. Additionally, glycoproteoform profiles of alpha‐1‐antichymotrypsin, identified through high‐resolution native mass spectrometry, revealed increased fucosylation and branching/LacNAc elongation.^432^ More importantly, the alterations in glycoproteoforms of alpha‐1‐antichymotrypsin induced by sepsis appear to remain stable after intensive care unit treatment, potentially serving as a prognostic indicator following intensive care.

In conclusion, investigating the changes in glycosylation, both in bacteria or viruses and in the host glycoproteins, is crucial for the effective diagnosis and treatment of diseases.

## 4 PERSPECTIVE

Protein glycosylation plays crucial roles in both physiological and pathological processes. The system‐wide analysis of the glycoproteomic code may offer significant prospects for accurate diagnosis and treatment of various diseases. The complexity of glycosylations imparts a wealth of information to glycoproteins; however, it is currently impossible to extract this information solely from the genetic code. Since the identification of the chemical properties of glycans, MS techniques have been widely employed to identify glycosylation patterns in various diseases.^433^ The objective of MS‐based clinical glycoproteomics is to investigate the relationship between disease and glycosylation‐encompassing glycoproteins, glycosites, and glycans‐on a large scale within a single experiment. Nevertheless, significant challenges persist, including the low abundance of glycosylations, the intricate structure of glycan chains, and the micro‐ and macro‐heterogeneity inherent in glycosylations. Fortunately, through the relentless efforts of researchers, the clinical glycoproteomic methodology has undergone significant advancements in recent years. These improvements encompass new enrichment materials, refined sample preparation techniques, revolutionary mass spectrometers, enhanced acquisition methods, and specialized software.

Our perspectives on the future development of clinical glycoproteomic methodologies encompass several key points. First, it is essential to establish a standardized clinical sample repository and implement rigorous management protocols for sample collection, transportation, storage, and data organization. This event is decisive for the reliability of subsequent findings. Second, we must develop standardized procedures for sample handling and establish quality evaluation criteria. For instance, high‐purity glycoproteins, recombinant glycoproteins, and synthetic IGPs should be utilized as reference materials. In addition, automated sample handling processes reduce the impact of human factors. Third, depending on the type of glycosylations being investigated, selecting the appropriate mass spectrometer, fragmentation mode, and data acquisition method is crucial. High‐resolution mass spectrometry, particularly in conjunction with suitable fragmentation modes and DIA techniques for glycoproteomics, represents a promising direction for the future. In addition, the development of in situ, real‐time, imaging MS technology to demonstrate the dynamic changes in glycosylation in clinical samples represents a major breakthrough in this field. Fourth, it is essential to develop both qualitative and quantitative software designed for the precise analysis of IGPs. The accurate identification and quantification of IGPs from large volumes of mass spectral data remains a significant bottleneck in this field. Glycoproteomics has led to the emergence of various commercial and academic software solutions that show promise for annotating IGPs derived from MS/MS data. However, substantial differences exist in the relative performance of these software tools. Future advancements may focus on enhancing accuracy through the integration of machine learning, artificial intelligence, and other innovative methodologies. Additionally, the establishment of a glycoproteomic data analysis and visualization platform is an important direction for lowering the entry barrier in this field. Fifth, necessary validation experiments represent the cornerstone for enhancing the reliability and utility of glycoproteomic analysis data. Currently, a significant portion of research remains focused on the stages of analysis and characterization, with a noticeable absence of essential verification outcomes. This gap leads to challenges in correlating discovered glycosylations with their functions. Finally, a thorough analysis of various molecular‐level data from clinical samples, such as the genome, transcriptome, proteome, glycoproteome, glycome, and metabolome, is essential. This multiomics integration strategy lays the groundwork for a comprehensive grasp of the mechanisms and functions within biological systems. The integration of multiomics data necessitates the use of statistics, machine learning, and other methods and technologies to process and analyze information across different data levels. This approach not only enhances our understanding of complex biological processes but also paves the way for advancements in personalized medicine and targeted therapies. New trends in multiomics data analysis include single‐cell multiomics analysis, ‌spatio‐temporal multiomics analysis, ‌ multiscale multiomics analysis, and so on. In addition, some critical considerations related to the design and execution of clinical glycoproteomics experiments should be noted.^434^ In summary, improved glycoproteomic methods are expected to invigorate the field and encourage a growing number of studies that investigate both fundamental and applied questions in clinical glycoproteomics.

Clinical glycoproteomics has shown considerable promise in differentiating between disease subtypes and predicting clinical outcomes across a range of human diseases, including brain diseases, CVDs, cancers, kidney diseases, and metabolic diseases. This innovative field holds the potential to enhance diagnostic sensitivity and precision, ultimately improving patient care and treatment strategies. However, realizing this potential remains a significant challenge. Moreover, while numerous glycoproteins with abnormal expression have been identified in various diseases, their specific functions and connections to disease progression remain poorly understood. This uncertainty regarding causality poses a significant challenge to the clinical glycoproteomic application. Our perspectives on the future development of clinical glycoproteomic applications encompass several key considerations. First, it is essential to establish standardized methods for the absolute quantification of glycoproteins, glycans, and IGPs in clinical samples. These methods should be characterized by their simplicity, speed, and cost effectiveness to facilitate their use in clinical testing. Encouragingly, we are actively working toward the establishment of a robust glycoproteomic measurement system. Second, the study of glycoproteomics in large cohorts and multicenter clinical samples is poised to become mainstream, with essential validation experiments being conducted. Third, cross‐disciplinary collaboration presents exciting new opportunities for clinical glycoproteomics. Innovations in areas such as new materials, visualization technologies, artificial intelligence, and multiomics integration are paving the way for advancements in this field. Fourth, addressing the challenges related to the clinical translation of glycosylation‐related biomarkers is crucial. These biomarkers have the potential to be more precise than those based on proteins alone. Nevertheless, the complexity of considering various factors, including proteins and glycan chains, necessitates the development of a comprehensive assay. This assay must seamlessly integrate both sets of information to facilitate clinical application. Finally, clinical glycoproteomics can significantly aid in the diagnosis of various diseases, research into underlying mechanisms, efficacy evaluation, target discovery, drug design, and the research and development of vaccines, among other applications.

In summary, the innovation of sample processing methods, advancements in mass spectrometry technology, the evolution of data analysis tools, and interdisciplinary collaboration are expected to substantially enhance the analytical capabilities of glycoproteomics. This progress will enable researchers to identify and quantify glycoproteins in complex biological samples, thereby facilitating the discovery and validation of disease biomarkers and providing valuable guidance for clinical decision‐making. Consequently, these findings will significantly advance the application of glycoproteomics in the field of personalized medicine.

## AUTHOR CONTRIBUTIONS

*Concept and design*: Yong Zhang and Lijun Zhao. *Data analysis and interpretation*: Yujia Wang and Kaixin Lei. *Manuscript writing*: Yujia Wang and Yong Zhang. *Final approval of manuscript*: all authors.

## CONFLICT OF INTEREST STATEMENT

The authors declare that they have no conflict of interest.

## ETHICS STATEMENT

No ethical approval was needed.

## Data Availability

All data and materials are available in the main text.
